# Anti-Inflammatory Function of Fatty Acids and Involvement of Their Metabolites in the Resolution of Inflammation in Chronic Obstructive Pulmonary Disease

**DOI:** 10.3390/ijms222312803

**Published:** 2021-11-26

**Authors:** Stanislav Kotlyarov, Anna Kotlyarova

**Affiliations:** 1 Department of Nursing, Ryazan State Medical University, 390026 Ryazan, Russia; 2 Department of Pharmacology and Pharmacy, Ryazan State Medical University, 390026 Ryazan, Russia; kaa.rz@yandex.ru

**Keywords:** COPD, fatty acids, inflammation, inflammation resolution, lipid mediators, specialized pro-resolving mediators, short-chain fatty acids

## Abstract

Lipid metabolism plays an important role in many lung functions. Disorders of lipid metabolism are part of the pathogenesis of chronic obstructive pulmonary disease (COPD). Lipids are involved in numerous cross-linkages with inflammation. Recent studies strongly support the involvement of fatty acids as participants in inflammation. They are involved in the initiation and resolution of inflammation, including acting as a substrate for the formation of lipid mediators of inflammation resolution. Specialized pro-inflammatory mediators (SPMs) belonging to the classes of lipoxins, resolvins, maresins, and protectins, which are formed enzymatically from unsaturated fatty acids, are now described. Disorders of their production and function are part of the pathogenesis of COPD. SPMs are currently the subject of active research in order to find new drugs. Short-chain fatty acids are another important participant in metabolic and immune processes, and their role in the pathogenesis of COPD is of great clinical interest.

## 1. Introduction

Chronic obstructive pulmonary disease (COPD) is one of the most important noncommunicable diseases, characterized by a variety of pulmonary and extrapulmonary clinical manifestations, based on local and systemic inflammation mainly due to long-term exposure to tobacco smoke components [1].

COPD is a clinically heterogeneous chronic disease. Moreover, this clinical heterogeneity has both pulmonary and extrapulmonary characteristics, which is the basis for phenotyping of patients [2]. The concept of phenotypes implies a search for specific clinical variants of a disease, united by common pathophysiological mechanisms, clinical characteristics, and impact on prognosis, which may be useful in selecting optimal therapeutic strategies. While for some diseases, the known phenotypes are not questioned by experts and clinicians. At the moment, there are no universally recognized phenotypes of COPD, which would fully meet the criteria of this term and would improve the results of treatment of all patients. The complexity of this situation is also due to the fact that the concept of phenotype implies more of an assessment of the clinical characteristics of the disease, without taking into account the underlying pathophysiological mechanisms. Accordingly, research is ongoing to find endotypes of the disease based on the commonality of impaired biological mechanisms.

Emphysema and chronic bronchitis are known to be two key disease phenotypes that were described long before the term COPD itself was coined. However, all the mechanisms that can lead to the development of emphysema are still the subject of debate.

Acute exacerbations of COPD make a major contribution to the clinical picture of COPD [3]. The frequency and severity of exacerbations influence disease progression and are associated with prognosis [4]. Bacterial colonization of the bronchi, local and systemic inflammation are considered important mechanisms associated with pulmonary and extrapulmonary clinical heterogeneity of the disease and the presence of comorbid pathology. Given the importance of exacerbations for the course of the disease and prognosis, some researchers have suggested a COPD phenotype with frequent exacerbations [5].

An important factor complicating the interpretation of COPD studies is that most studies do not take into account the heterogeneity of the disease. This may be both a cause and a consequence of a lack of understanding of the mechanisms underlying the development and heterogeneous course of COPD. In this regard, it should be noted that there is convincing evidence that the course of COPD is related to lipid metabolism.

Lipids play an important role in lung function. The lungs are known for their unique lipid biology, which is involved in lung structure and respiratory function. The lipid landscape of the lungs is very diverse. The importance of lipid balance is well demonstrated by surfactant, the deficiency of which causes severe impairment of respiratory function. Saturated fatty acids such as palmitic and stearic acids are components of pulmonary surfactant [6]. The complexity and lack of study of lipid metabolism are well illustrated by the link between not only body weight and COPD phenotypes, but also the prognosis of the disease.

Studies in recent years have convincingly demonstrated the involvement of lipids in inflammation. Smoking has been shown to disrupt the normal processes that maintain lipid homeostasis in the lungs, which may be part of the pathogenesis of COPD [7]. It should be noted that cigarette smoke contributes to an oxidant/antioxidant imbalance due to exogenous reactive oxygen species (ROS). Exogenous, as well as endogenous ROS produced by inflammation and mitochondrial dysfunction, may be involved in the oxidation of various biomolecules, including lipids, leading to epithelial cell damage and death, which is one of the key factors in the development of COPD [8].

The data accumulated in recent years have significantly expanded the understanding of the role of lipids as participants in various links of inflammation [9]. Inflammation is a universal mechanism that responds to a variety of tissue damage. The process of inflammation is believed to have not only an initialization phase, but also a resolution phase, which plays an important role in ensuring tissue immune homeostasis. And, as was found in a number of studies, the resolution phase of inflammation is active and mediated by a number of biological factors. In addition, it is also proposed to distinguish the “post-resolution” phase, which is also anti-inflammatory, is regulated by macrophages and dendritic cells and is necessary for the subsequent immune response due to its influence on adaptive immunity [10,11,12].

Given the importance of lipid metabolism for lung function, the purpose of this review is to discuss the involvement of fatty acids and their lipid metabolites as mediators of inflammation initiation and resolution in the development and progression of COPD.

## 2. Long-Chain Fatty Acids

Data accumulated in recent years have improved our understanding of the functions of lipids in the different phases of inflammation. There is increasing evidence that lipids are not simply a source of energy or structural material for cells, but are actively involved both in the initiation and maintenance of inflammation, such as prostaglandins and leukotrienes, and are also mediators of the highly organized resolution phase of inflammation.

**Figure 1:**
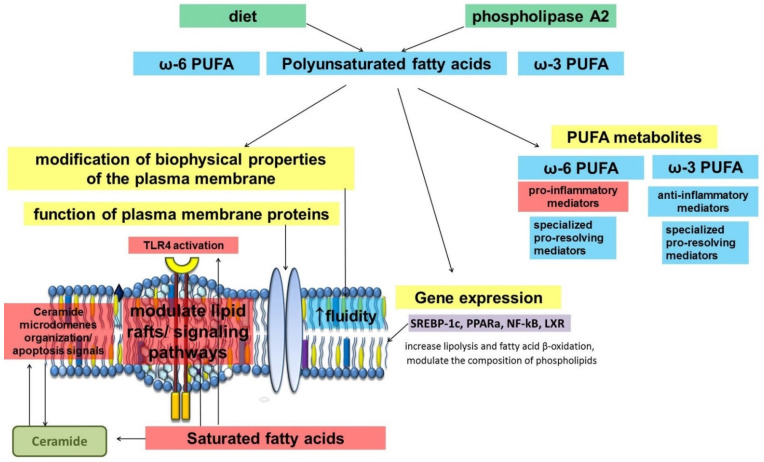
Scheme of fatty acids involvement in pro- and anti-inflammatory mechanisms.

Analysis of the role of fatty acids in inflammation demonstrates the diversity of their function (Figure 1) [13,14]. As part of the phospholipids of plasma membranes, fatty acids can influence their structure and function [15]. The saturation and length of the alkyl chain are important. The biophysical properties of the plasma membrane and the stability of lipid rafts, and consequently the function of some membrane proteins, can be related to the chemical structure of fatty acid residues. Available data suggest that unsaturated fatty acids contribute to a decrease in lipid ordering and lipid raft stability. It has been suggested that this may lead to anti-inflammatory effects, given that lipid rafts are considered to be platforms for the assembly and function of many signaling pathways.

Regulation of membrane biophysical properties is an important tool for the function of many membrane proteins. Lipid ordering can influence the possibility of conformational changes in proteins as they perform their functions. The composition of fatty acids in membrane phospholipids can provide a balance between optimal membrane fluidity to allow the necessary conformational changes of proteins and the viscosity required for their localisation in the membrane. For example, by altering the biophysical properties of the membrane, ω-3 PUFAs can enhance the activity of transient receptor potential vanilloid 4 (TRPV4) [16].

TRPV4 has many functions in lung cells and is involved in the pathogenesis of COPD. It is involved in the control of epithelial and endothelial permeability, as well as causing contraction of bronchial smooth muscles and taking part in autoregulation of mucociliary transport [17]. Adult TRPV4^−/−^ mice exhibit emphysema-like changes in the lungs [18]. Recently, TRPV4 activation by ω-3 PUFAs has been shown to be associated with endothelial protective mechanisms, given that TRPV4 regulates endothelium-dependent vascular relaxation associated with nitric oxide release under physiological conditions [19].

Thus, the effect of ω-3 PUFAs on the biophysical properties of membranes and the function of membrane proteins may be related to the features of the disease course and is a promising area for future research.

Smoking is known to cause decreased fluidity of plasma membranes of alveolar macrophages in rats [19,20]. It has been suggested that smoking-induced lipid peroxidation primarily affects unsaturated fatty acids in membrane phospholipids, which is reflected in the biophysical properties of membranes [19,21].

In addition to their role in the function of membrane proteins and the regulation of their signaling pathways through altering the biophysical properties of plasma membranes, fatty acids can directly stimulate receptors such as toll like receptor 4 (TLR4) [22]. TLR4 is an important participant in the immune response in COPD, as its function is to detect lipopolysaccharide (LPS) of Gram-negative bacteria. Only saturated fatty acids can activate TLR4, whereas unsaturated fatty acids do not [23,24,25]. These data emphasize the differential role of saturated and unsaturated fatty acids in inflammation [26].

Another mechanism of ω-3 polyunsaturated fatty acids (PUFAs) involvement in immunity is their putative links to histone acetylation. Prenatal intake of polyunsaturated fatty acids from fish oil and olive oil has been shown to affect histone acetylation of genes involved in adaptive immunity [27,28].

COPD is characterized by impaired fatty acid metabolism in the stable course and in exacerbations [29]. These changes affect both fatty acids in phospholipids of plasma membranes and free fatty acids.

Modification of fatty acid composition of plasma membranes of leukocytes of patients with COPD is characterized by an increase in the relative content of saturated lauric, palmitic, stearic acids, unsaturated arachidonic acid and depletion of the pool of linoleic, docosatetraenoic, eicosapentaenoic, docosahexaenoic acids [30]. A decrease in ω-3 PUFAs is found in the mitochondrial membranes of leukocytes, which may affect their function [31].

Disorders of fatty acid composition in COPD have also been found in erythrocyte membranes [32,33,34]. In patients with COPD and chronic bronchitis, accumulation of arachidonic acid and deficiency of eicosapentaenoic acid in plasma membranes of erythrocytes were found. Moreover, this imbalance is more pronounced in patients with COPD than in those with chronic bronchitis [35]. In addition to the accumulation of arachidonic acid, COPD patients were found to accumulate pentadecanoic acid, docosatetraenoic acid, stearic acid, ecosanic acid, and decrease in linoleic acid, eicosapentaenoic acid, and docosapentaenoic acid. The detected imbalance corresponded to increased accumulation of ω-6 PUFAs. In addition, an increase in the (20:4, n-6)/(20:3, n-6) ratio was noted in COPD patients, which may indirectly indicate the activation of delta-5 desaturase, and intensification of substrate biosynthesis for eicosanoids formation [35]. Interestingly, in very severe COPD, in addition to increased membrane content of arachidonic acid, phospholipids and cholesterol levels in erythrocyte plasma membranes also increase, which have a negative correlation with forced expiratory volume in one second (FEV1%) [33]. These data indicate a systemic nature of the changes, which may be associated with the progression of the disease and its extrapulmonary clinical heterogeneity.

Patients with COPD in the stable phase have lower levels of free alpha-linolenic acid, linoleic acid and eicosapentaenoic acid in sputum [29,32]. At the same time, higher levels of free arachidonic acid and docosapentaenoic acid were observed during acute exacerbation of COPD compared with stable COPD [33].

Plasma levels of eicosapentaenoic acid and docosahexaenoic acid, which included different lipid classes such as cholesterol esters, phosphatidylcholine and lysophosphatidylcholine, were found to decrease in the blood plasma of smokers with mild to moderate COPD [36]. At the same time, serum levels of monounsaturated fatty acids (MUFA, 16:1, 18:1), such as cholesterol esters 16:1, diacylglycerols 18:1/18:1 and phosphathidylcholines 16:1/18:1 were increased in the serum of smokers, especially those with mild to moderately severe COPD [36]. PUFAs, as already noted, are sensitive to oxidative damage, and, accordingly, a decrease in their levels can be considered as a marker of oxidative stress [37,38].

In addition to abnormalities in fatty acid levels in healthy smokers and smokers with mild to moderate COPD, changes in serum eicosanoids levels were also found. These changes in smokers included increased levels of 11,12-dihydroxy-5Z,8Z,14Z-eicosatrienoic acid (11,12-DHET), 4,15-dihydroxy-5Z,8Z,11Z-eicosatrienoic acid (14,15-DHET) (dihydroxyeicosatrienoic acids) and 15-Hydroxy-5Z 8Z,11Z,13E-eicosatetraenoic acid (15-HETE) and reduced levels of 9-Hydroxy-10E,12Z-octadecadienoic acid (9-HODE) and 13-hydroxy-9Z,11E-octadecadienoic acid (13-HODE) (hydroxyoctadecadienoic acids) [36]. Changes in lipid profiles have demonstrated associations with clinical characteristics of COPD, e.g., PUFAs showed positive correlations with lung function [36].

It is known that the fatty acid composition of erythrocyte plasma membranes is influenced not only by smoking. It was shown that obese children had higher levels of ω-6 polyunsaturated fatty acids (mainly arachidonic acid) and lower levels of monounsaturated fatty acids, resulting in an increased saturated fatty acid ratio (SFA)/MUFA [39].

These and other data suggest a presumed important role for fatty acids in inflammation in COPD and, consequently, in the development of pulmonary and extrapulmonary clinical manifestations of the disease.

In addition to their involvement in inflammation, fatty acids are an important source of energy in COPD. The decrease in plasma free fatty acids in patients with COPD [40], may be related to the increased need in these patients for high-energy substrates, due to the need to maintain inflammation and more intensive respiratory work. In distal airway epithelial cells, acute exposure to cigarette smoke results in increased carnitine palmitoyltransferase (CPT1A) activity and increased β-oxidation of fatty acids. This leads to a switch of cellular energy metabolism from glucose, which is the main energy source, to lipids [41,42]. Given that carbohydrate catabolism is accompanied by the formation of large amounts of carbon dioxide, the use of fatty acids as an energy substrate contributes to less CO_2_ production. Given the impaired CO_2_ excretion in COPD, using fatty acids as an energy source may reduce some of the negative effects of the disease related to muscle dysfunction and shortness of breath [43,44]. And a diet low in carbohydrates but with the addition of medium chain triglycerides and predominantly monounsaturated fatty acids in the diet help improve pulmonary function in patients with COPD [45].

Interestingly, but exposure of lung endothelial cells to cigarette smoke decreases β-oxidation of fatty acids, which leads to increased ceramide synthesis and endothelial cell apoptosis [46]. Endothelial cell apoptosis is one of the key events in the development of emphysema. It has been shown that these processes may be associated with elevated levels of ceramides in the lungs of COPD patients, which are regarded as a marker of the disease. It should be noted that ceramides may have several pathways of formation, including those associated with the action of acid sphingomyelinase and also as a result of synthesis involving palmitate on exposure to cigarette smoke [46]. Ceramides form lipid rafts in plasma membranes with specific biophysical properties on which certain apoptosis-related signaling pathways are organized. The physical properties of ceramides are affected by the length of the fatty acid chain [47,48,49,50]. Moreover, fatty acids with 16-24 carbon atoms are most frequently included in the ceramides of plasma membranes, due to the fact that they are the least polar and the most hydrophobic [50,51].

In contrast to ceramides, high levels of ω-3 PUFAs in plasma are associated with decreased progression of emphysema [52].

Unsaturated fatty acids are involved in inflammation not only because of their biophysical properties. They can act as precursors for the formation of many lipid mediators associated with inflammation. For example, arachidonic acid is a substrate for the synthesis of prostaglandins (PG) and leukotrienes (LT), which are involved in the initiation of acute inflammation [53,54]. However, arachidonic acid is a precursor for the formation of lipoxin A4 (LXA4), which is considered to be an important participant in the resolution of inflammation [55,56,57,58]. In this regard, arachidonic acid demonstrates a differential pattern of involvement in inflammation. Interestingly, exposure to arachidonic acid in the experiment resulted in increased release of IL-6 and CXCL8 from fibroblasts, and the release of IL-6 and CXCL8 was reduced in COPD compared with patients without COPD. The lower production of cytokines in COPD compared with pulmonary fibroblasts without COPD suggests differences in the involvement of arachidonic acid in inflammation in different diseases [59].

Thus, free fatty acids and fatty acids in the phospholipids of plasma membranes can be considered as depots for mediator biosynthesis. In response to tissue damage, unsaturated fatty acids can be mobilized by phospholipase A2 from phospholipids for subsequent conversion into lipid mediators [60].

## 3. Specialized Pro-Resolving Mediators

All aspects of the delicate balance of lipid mediator involvement in inflammation have yet to be studied, but it is already known that members of the family of lipid mediators, which have been named “specialized pro-resolving mediators” (SPMs), play a key role in the active resolution of inflammation [61].

**Figure 2:**
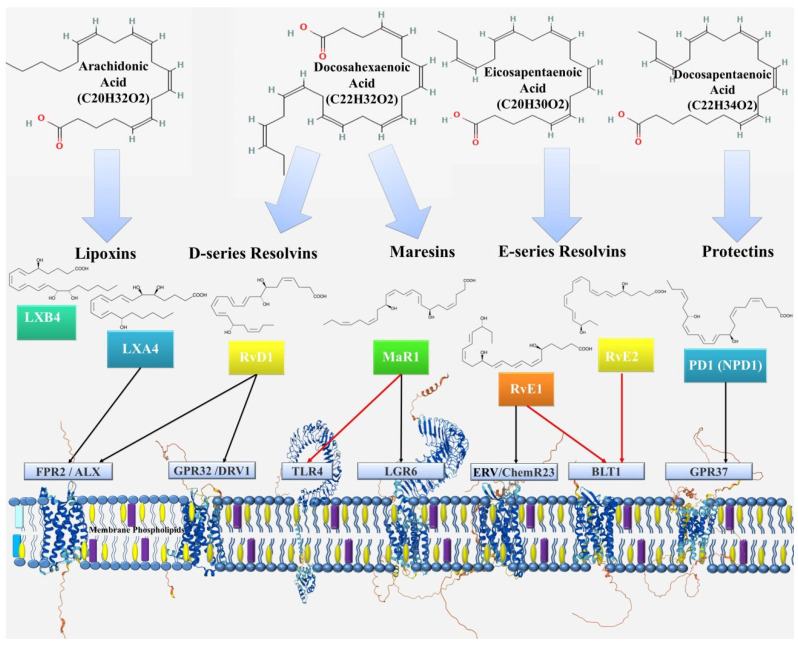
Scheme of formation and action of specialized pro-solving mediators. Red lines indicate receptor inhibition, black lines indicate receptor activation. Abbreviations: lipoxin A4 (LXA4); lipoxin B4 (LXB4); resolvin D1 (RvD1); resolvin E1 (RvE1); resolvin E2 (RvE2); maresin 1 (MaR1); protectin D1 (PD1) or neuroprotectin D1 (NPD1); N-formyl peptide receptor 2/ALX receptor (FPR2/ALX); G protein-coupled receptor 32/resolvin D1 receptor (GPR32/DRV1); Toll-like receptor 4 (TLR4); leucine-rich repeat containing G protein–coupled receptor 6 (LGR6); series E resolvin receptor/chemerin receptor 23 (ERV/ChemR23); leukotriene B4 receptor 1 (BLT1); G-protein coupled receptor 37 (GPR37).

This class of endogenously produced bioactive lipids is diverse and includes Lipoxins, Resolvins, Protectins, and Maresins, which are formed enzymatically from ω-3 and ω-6 PUFAs, such as arachidonic acid, eicosapentaenoic acid, docosahexaenoic acid and docosapentaenoic acid (Figure 2).

Lipoxins are synthesized from arachidonic acid, E-series resolvins from eicosapentaenoic acid, D-series resolvins and protectins, and maresins from docosahexaenoic acid. Thus, PUFAs, are an important source of not only proinflammatory but also anti-inflammatory mediators. The factors that provide this balance are still largely unclear, but their better study may be the key to understanding the pathogenesis of many diseases.

The data available to date highlight the significant role of SPMs in inflammation, which is provided by the regulation of numerous downstream signaling pathways [10]. 

In addition, the proresolving effects of some SPMs are, in part, related to their ability to regulate redox states in cells by inhibiting oxidative stress. Moreover, this protection is related not only to the reduction of ROS production but also through the enhancement of several natural antioxidant defences such as modulation of superoxide dismutase, heme oxygenase-1 and nuclear factor erythroid 2-related factor 2 expression [62].

Taking into account the information about impaired resolution of inflammation in COPD, the role of lipid mediators is of great clinical interest. Analysis of the known data suggests that lipid mediators are involved in inflammation in a coordinated manner. The appearance of lipid mediators of inflammation (leukotrienes and prostaglandins) are coordinated with neutrophil recruitment. Leukotriene B4 (LTB4), which is a chemoattractant [63,64], is involved in neutrophil recruitment [61,65]. Prostaglandin (PGE2) then promotes the switch of biosynthesis from LTB4 involving 5-lipoxygenase (5-LO), to LXA4 involving 15-LO, which leads to a decrease in tissue infiltration by neutrophils [66,67].

Thus, lipid mediators demonstrate a coordinated role in ensuring the phase change of inflammation. At the same time, SPMs affect a decrease in the secretion of proinflammatory cytokines, contribute to an increase in the production of anti-inflammatory cytokines, through switching macrophages to the M2 phenotype, and also increase phagocytosis, which is important, given that tobacco smoke stimulates macrophages proinflammatory.

### 3.1. Lipoxins

**Figure 3:**
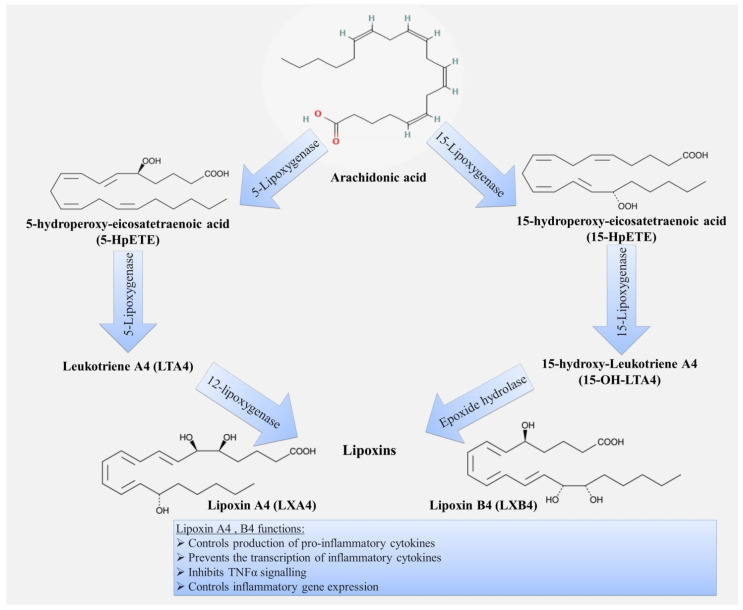
Scheme of biosynthesis and functions of lipoxins.

Lipoxins, the first identified class of SPMs, are synthesized from arachidonic acid by the sequential action of lipoxygenase (LOX) enzymes, including 5-, 12- and 15-LOX (Figure 3).

Lipoxin A4 (LXA4) and lipoxin B4 (LXB4), and their epimers: 15-epi-LXA4 and 15-epi-LXB4 have been identified so far. The structure of lipoxins is based on their origin from ω-6 arachidonic acid and includes three hydroxyl residues and four double bonds, which distinguishes them from other SPMs originating from ω-3 fatty acids. Thus, arachidonic acid, which is a metabolite for the synthesis of both pro- and anti-inflammatory mediators, is at the crossroads of the inflammatory pathways.

The receptor through which the lipoxins LXA4 and 15-epi-LXA4 exert their action is FPR2 (also called ALX receptor, ALX/FPR, ALX/FPR2, and FPRL1). ALX/FPR2 is a receptor with seven transmembrane domains and is expressed in airway epithelial cells as well as other cells involved in inflammation, including neutrophils, mast cells, monocytes, macrophages, lymphocytes, and dendritic cells [68,69,70,71,72,73,74,75,76].

LXA4 exhibits multiple anti-inflammatory relationships. It promotes inhibition of chemotaxis, transendothelial, and transepithelial migration of neutrophils [61,77,78], and inhibits their interaction with epithelial cells [61,71,77,79]. In addition, LXA4 stimulates monocyte chemotaxis and adhesion [80] and increases the uptake of apoptotic neutrophils by macrophages [81]. These actions promote clearance of apoptotic leukocytes by macrophages at the site of inflammation [81,82,83].

LXA4 plays a role in bronchial epithelial repair by triggering the migration and proliferation of epithelial cells [71,83,84]. The effects of LXA4 in restoring the epithelium and airway surface liquid are mediated by apical release of ATP and activation of the purine receptor P2Y11 [83,85].

The anti-inflammatory effect of LXA4 also consists in the suppression of IL8 production by leukocytes and bronchial epithelial cells [83,86,87,88,89].

LXB4, as well as LXA4, can inhibit the migration of polymorphonuclear neutrophils stimulated by LTB4 and also weaken the adhesion of polymorphonuclear neutrophils to endothelial cells mediated by P-selectin [78].

In addition, aspirin-triggered lipoxin A4 (ATLs) can inhibit proliferation and migration of endothelial cells, disrupting angiogenesis [90]. 15-epi-LXA4 also increases the resolution of pulmonary inflammation by promoting neutrophil apoptosis [91].

COPD has been shown to be characterized by decreased lipoxin production. Decreased concentrations of LXA4 in induced sputum have been shown in patients with COPD compared with healthy individuals [92,93]. A decrease in LXA4 was also found in the exhaled breath condensate of moderate to severe COPD patients [94]. This may be one of the causes of persistent inflammation in the airways.

In addition, COPD patients have decreased levels of lipoxin receptor in alveoli, which may explain the persistence of inflammation in COPD. At the same time, asymptomatic smokers were found to have increased levels of FPRL1 in alveolar walls, which may be an adaptive anti-inflammatory mechanism [95]. In addition, in smokers, the number of cells with FPRL1 correlated with airflow obstruction, FEV1% [95].

Interestingly, LXA4 may be associated with the regulation of reverse cholesterol transport through a dose-dependent increase in ATP binding cassette transporter A1 (ABCA1) and Liver X receptor alpha (LXRa) expression in «foam cells» derived from THP-1 macrophages [96]. These findings significantly broaden the view on the function of LXA4, considering the negative effect of cellular cholesterol accumulation on inflammation. The tobacco smoke-induced decrease in ABCA1 expression and functional activity in lung macrophages is associated with impaired reverse cholesterol transport and their inflammatory activation. Thus, increased ABCA1 expression mediates the anti-inflammatory role of LXA4.

The effect of lipoxins on cholesterol metabolism may be mediated by increased expression of another member of the large family of ABC transporters, Abcb11, through a post-transcriptional and post-translational mechanism involving MAPK p38 activity [97]. Abcb11 is involved in lipid homeostasis through regulation of biliary lipid secretion.

It has also been shown that decreased serum LXA4 levels correlate with the development of metabolic syndrome, therefore, assessment of LXA4 levels can be used for early detection and prevention of metabolic syndrome [98].

These and other findings have expanded the understanding of LXA4 in the pathogenesis of COPD from the perspective of the analysis of pulmonary and extrapulmonary clinical heterogeneity of the disease and comorbid relationships. Given that the most significant comorbid conditions of COPD include cardiovascular disease and above all those associated with atherosclerosis, understanding the role of lipid mediators brings the discussion of the problem to a new level. Recent data on deficient production of 15-epi-LXA4 in patients with peripheral arterial disease suggest a protective role of LXA4 in atherogenesis [99]. These data reinforce the importance of lipoxins, given the frequent comorbid links between COPD and peripheral atherosclerosis.

### 3.2. Resolvins

**Figure 4:**
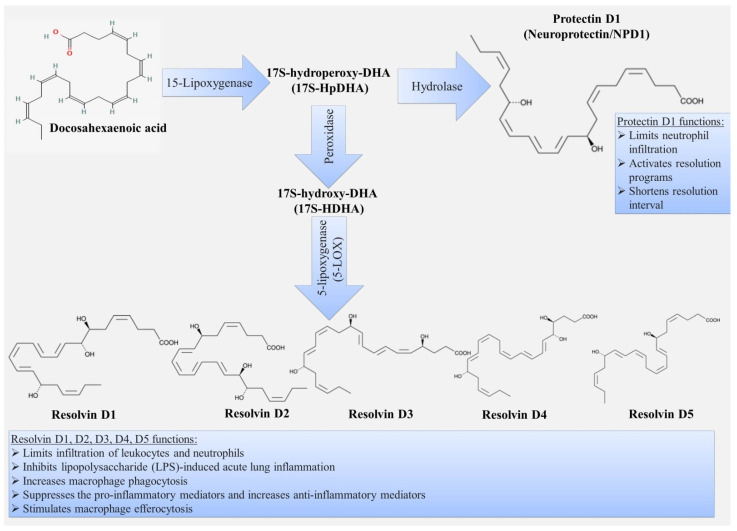
Scheme of biosynthesis and functions of D-series resolvins and protectins.

**Figure 5:**
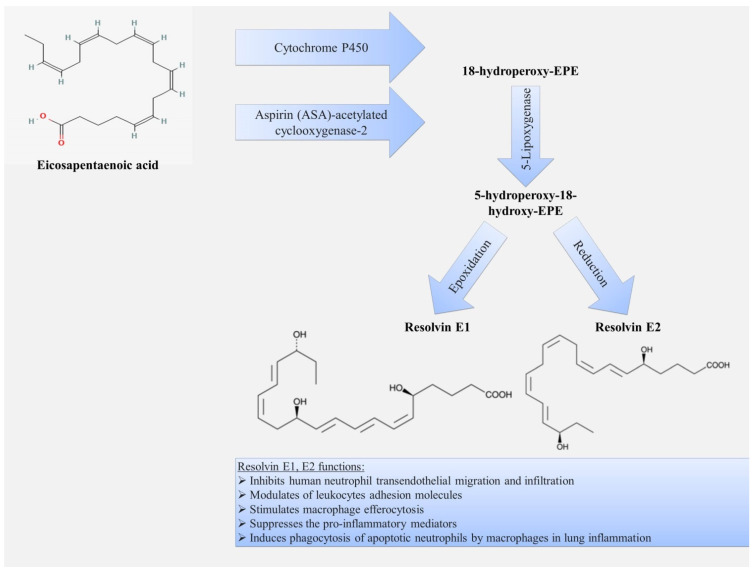
Scheme of biosynthesis and functions of E-series resolvins.

Resolvins are small lipid molecules that are synthesized from ω-3 PUFAs such as eicosapentaenoic acid and docosahexaenoic acid. The term “resolvins” itself reflects their role as a key participant in the resolution phase of acute inflammation. Resolvins belonging to the D series (formed from docosahexaenoic acid) (Figure 4) and E series (formed from eicosapentaenoic acid) (Figure 5) and epimers of these classes formed when aspirin inhibits cyclooxygenase have now been identified. D-series resolvins include RvD1,2,3,4,5,6 [10], and E-series resolvins include RvE1,2,3,4 [100].

The receptors for RvD1 are the lipoxin receptor FPR2/ALX and DRV1 (also known as GPR32) [101], but activation of the GPR32 receptor requires lower concentrations of RvD1 than are necessary to activate FPR2/ALX [102,103]. DRV1 is expressed on neutrophils, lymphocytes, monocytes, and macrophages [69,101]. In addition to RvD1, this receptor is also activated by other ligands, such as AT-RvD1, RvD3, AT-RvD3, and RvD5 [69,104,105,106,107]. It is believed that RvD1 interacts with DRV1 during periods of homeostasis and via ALX/FPR2 during the resolution of inflammation [69].

At present, there are numerous data confirming the involvement of resolvins in the regulation of inflammation. It has been shown that RvD1 is a powerful regulator of neutrophil activity, controlling their migration through the endothelium [107,108]. In addition, RvD1 reduces inflammation by inhibiting the release of proinflammatory cytokines induced by LPS in macrophages [109,110,111]. By acting on human alveolar macrophages, RvD1 and RvD2 reduce the production of inflammatory mediators such as interleukin-6 (IL-6) and tumor necrosis factor-α (TNF-α), while promoting the production of anti-inflammatory cytokines. These resolvins are involved in alternative M2 activation of macrophages and can also attenuate the resulting effects of oxidative stress induced by cigarette smoke [112]. In addition, RvD1 and RvD2 enhance phagocytosis of apoptotic cells by macrophages, which is impaired by smoking [113,114,115,116]. The anti-inflammatory effect of RvD2 can also be realized through modulation of NF-κB signaling pathways [112].

In experiments on mouse models with long-term exposure to cigarette smoke, RvD1 has been shown to reduce inflammation and emphysema development [117]. This is associated with decreased formation of proinflammatory mediators, decreased neutrophilic inflammation, and increased production of the anti-inflammatory cytokine IL-10. RvD1 promoted efferocytosis of neutrophils and alternative activation of M2 macrophages [118]. An epimeric aspirin-triggered RvD1 showed similar results in experiments on mice with cigarette smoke-induced emphysema [119]. In addition, RvD1 reduces apoptosis and inflammation of alveolar epithelial type 2 cells caused by LPS exposure [120].

During chronic *P. aeruginosa* lung infection in an experimental mouse model, RvD1 regulated the expression of Toll-like receptors in macrophages, their downstream genes and microRNA (miR)-21 and 155, which led to a decrease in inflammatory signaling. In in vitro experiments, RvD1 demonstrated similar actions, enhancing phagocytosis of *P. aeruginosa* by neutrophils and macrophages [121].

At the same time, in COPD patients the concentration of RvD1 was reduced in bronchoalveolar lavage fluid and serum [112]. Exogenous administration of RvD1 can significantly reduce the number of neutrophils induced by cigarette smoke exposure, as well as reduce inflammation, oxidative stress manifestations and cell death [10,112,122].

Another member of the resolvins, RvD2 also promotes alternative M2 activation of monocyte-derived macrophages and prevents M1 polarization when exposed to cigarette smoke extract [112].

The best-known E series resolvins are resolvin E1 (RvE1) and resolvin E2 (RvE2). The receptor for this series of resolvins is the resolvin E series receptor (ERV), which is also known as chemokine-like receptor 1 (CMKLR1) and chemerin receptor 23 (ChemR23) [123]. ERV is widely present in various lung cell types, including airway epithelial cells as well as cells of the immune system, including neutrophils, monocytes, macrophages, and dendritic cells [69,70,124,125,126,127,128,129,130].

Resolvin E1 demonstrates an anti-inflammatory effect that consists in decreasing the recruitment of neutrophils, by inhibiting their transepithelial and transendothelial migration [124,131,132,133,134,135]. Another mechanism is the stimulation of efferocytosis of apoptotic neutrophils by macrophages [133,134], and inhibition of proinflammatory cytokine release [136,137]. Studies have shown that the implementation of the resolution phase of inflammation by RvE1 is mediated by its effect on migration and activation of the monocyte-macrophage system, through its specific binding to two types of receptors, ChemR23 and LTB4 receptor 1 (BLT1) [138,139,140].

In addition, RvE1 stimulates the expression by apoptotic leukocytes of the chemokine receptor CCR5. Thus, RvE1 demonstrates anti-inflammatory activity and promotes the resolution of inflammation. A study in a mouse model of pneumonia showed that RvE1 reduces the levels of several proinflammatory chemokines and cytokines in the lungs and improves survival [141].

Resolvins may also be involved in atheroprotection. RvD2 has been shown to be involved in the regulation of nitric oxide production, through which, as well as direct modulation of leukocyte adhesion receptor expression, it reduces leukocyte-endothelial interaction [140]. In addition to nitric oxide, RvD2 stimulates the release of prostacyclin from vascular endothelial cells [142,143].

RvE3 is also a potent inhibitor of polymorphonuclear leukocyte chemotaxis in vitro [144], in addition to this it also reduces allergic airway inflammation through the IL-23/IL-17A pathway, suggesting promise for this resolvin for asthma treatment [145]. These data are of interest given the frequent combination of COPD and asthma.

In addition, recently identified RvE4 is a potent stimulator of efferocytosis of senescent erythrocytes and apoptotic M2 neutrophils by macrophages [100,146].

Together, these data indicate an important and diverse role of resolvins in the resolution of inflammation. Disruption of their regulation may be part of the pathogenesis of COPD, and their synthetic analogues can be considered as promising means for treatment.

### 3.3. Protectins

Protectins (PDs), another member of the family of specialized pro-resolution mediators, are synthesized from two ω-3 polyunsaturated fatty acids, such as docosahexaenoic acid (DHA) and docosapentaenoic acid (DPA) (Figure 4) [147]. According to their chemical structure, they are E,E,Z-docosatrienes, because they have three conjugated double bonds located between the 10th and 17th carbon atoms. There are a total of 6 double bonds in the protectin molecule. The PD1 biosynthesis pathway begins with the enzymatic conversion of a fatty acid by 15-lipoxygenase (ALOX15) to 17S-hydroperoxy-DHA and then by enzymatic apocsidation to 16S,17S-epoxy-DHA, which after enzymatic hydrolysis is converted to 10R,17S-dihydroxy-docosa-4Z,7Z,11E,13E,15Z,19Z-hexaenoic acid (10R,17S-DT) or PD1. The subsequent products of PD1 metabolism have not been studied in vivo, but there are reports of a metabolite called 22-OH-PD1, which also exhibits powerful anti-inflammatory activity [148].

PD1 was first described in brain and retinal tissues as neuroprotectin D1 (NPD1), which was considered to be a mediator of protection against oxidative stress [149,150]. It was later found that PDs are formed in many tissues and have different functions. PD1 has been found in human lung tissue and exhaled breath condensate, in inflammatory exudate, in peripheral blood, and in a wide range of other cells and tissues.

Several types of protectins are distinguished-PD1 or NPD1, PD1-d5, 17(R)-PD1 and PDX, as well as conjugated protectins such as PCTR1, PCTR2 and PCTR3. Proteins have an anti-inflammatory effect by acting on the GPR37 receptor, also called PAELR (Parkin-associated endothelin receptor-like receptor) [147]. Protectins differ from one another in the severity of their anti-inflammatory effect, which is explained by differences in the stereochemistry of the molecules, for example the R-epimer PD1 is more active than the S-epimer PD1 [147,151].

The anti-inflammatory effects of PD1 include inhibition of neutrophil migration [152], reduction of TNF-α and interferon (IFN)-γ production by neutrophils [153]. In addition, it regulates CCR5 expression in neutrophils [154] and stimulates macrophage phagocytosis and efferocytosis [61,105,155], as well as reducing angiogenesis and promoting epithelial barrier integrity [11,61,156,157].

Thus, protectins are of research and clinical interest, and their role in the pathogenesis of COPD requires further research.

### 3.4. Maresins

**Figure 6:**
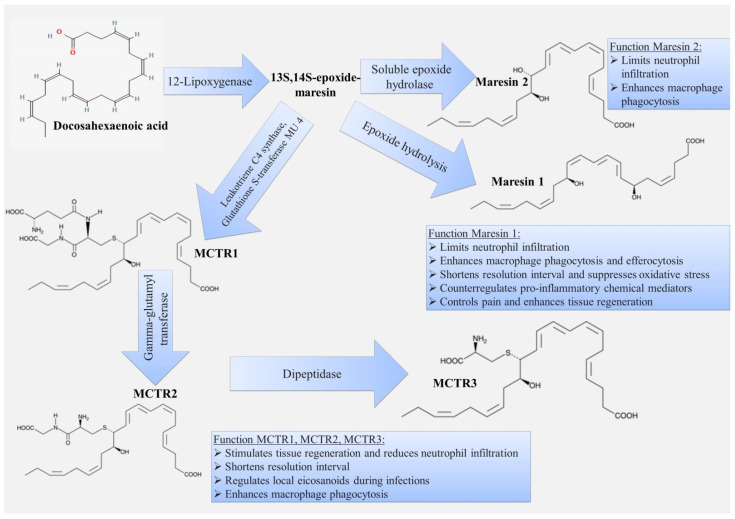
Scheme of biosynthesis and functions of maresins.

Maresins (MaRs), other members of SPMs, are synthesized from ω-3 docosahexaenoic acid (DHA) (Figure 6) [158]. Several types of maresins are distinguished-MaR1, MaR2, MaR1-d5, MaR2-d5, as well as maresin conjugate in tissue regeneration (MCTR), such as MCTR1, MCTR2, MCTR3. The formation of certain types of maresins depends on enzymes, e.g., epoxide hydrolysis is the key enzyme for conversion to MaR1 [159], soluble epoxide hydrolase for MaR2 [160], leukotriene C4 synthase and glutathione S-transferase MU 4 for MCTR1, gamma-glutamyltransferase for MCTR2 and dipeptidase for MCTR3 [161,162,163].

MaR1 was the first identified maresin and is described as a DHA product formed by macrophage cultures derived from human monocytes [158]. Its biosynthesis is initiated by a lipoxygenation process (the key enzyme is 12-lipoxygenase) at carbon-14 position, which introduces oxygen into the molecule. A 13S, 14S-epoxide-maresin intermediate is formed, which is further converted to one of the maresins by enzymatic transformations [161]. MaR1 and MaR2 share the chemical formula, C22H32O4, but differ in structure (position of the hydroxyl group at the 7 and 13 positions, respectively) and molecular configuration. As well as in their chemical structure, they have similarities and differences in the functions they perform. For example, MaR1 and MaR2 limit the recruitment of polymorphonuclear leukocytes and enhance macrophage phagocytosis and efferocytosis [160,164,165,166]. MaR1 contributes to decreased levels of proinflammatory cytokines in a mouse model of sepsis, such as IL-6, TNF-α, and IL-1β [161]. MaR1 is additionally involved in pain regulation [167] and also protects against lung damage by inhibiting oxidative stress, which can be partially explained by activation of the Nrf-2-mediated HO-1 signaling pathway [168].

MaR1 is considered an activator for leucine-rich repeat containing G protein-coupled receptor 6, which is expressed in phagocytes and which enhances phagocytosis and efferocytosis [169,170]. In addition, MaR1 can participate in the regulation of inflammation by decreasing TLR4 activation [171].

MCTR1, MCTR2, and MCTR3 have been studied to a lesser extent, but there is information on their role in tissue regeneration and regulation of neutrophil infiltration [161]. In a mouse model, MCTR1 accelerates the resolution of inflammation induced by LPS stimulation through M2 polarization of resident alveolar macrophages [172]. In addition, it contributes to a decrease in the production of inflammatory cytokines such as TNF-α, IL-1β, and IL-6. In an LPS-induced sepsis model in mice, it also contributes to the reduction of lung endothelial glycocalyx damage through the ALX/SIRT1/NF-kB/HPA pathway [173].

Thus, SPMs are a new promising direction for the study of COPD pathogenesis and search of new tools for treatment.

## 4. Participation of Fatty Acids in Immunometabolic Reprogramming of Macrophages

Macrophages are important participants and regulators of inflammation in COPD. The lungs have both their own population of alveolar macrophages and cells differentiated from blood monocytes. Macrophages are differentially involved in inflammation, demonstrating multiple functions related to their functional phenotype. The polarization of macrophages is related to their metabolic profile and is characterized by different production of biological factors involved in inflammation. The best-known are M1 and M2 (subtypes M2a, M2b, M2c, M2d) phenotypes of macrophages, which have pro- and anti-inflammatory functions, respectively. M1 macrophages are called “classically activated (proinflammatory) macrophages”. They produce high levels of proinflammatory cytokines such as TNF-α, IL-1ß, IL-6, IL-12, and also have strong bactericidal properties. M2 macrophages, in addition to producing anti-inflammatory factors such as IL-10, participate in tissue remodeling and are called “alternatively activated macrophages” [174,175,176].

Studies in recent years have shown that this classification is very simplistic, but it may be useful for the purpose of understanding the differentiated role of macrophages in inflammation.

**Figure 7:**
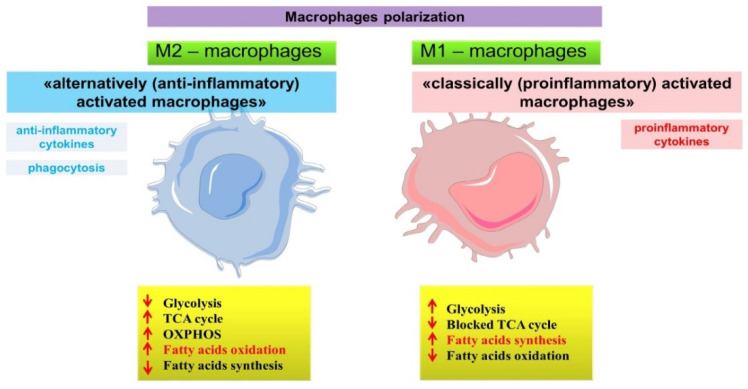
Scheme of immunometabolic reprogramming of macrophages involving fatty acids.

Interestingly, the polarization of macrophages is related to their metabolic reprogramming, including the differential nature of fatty acid utilization (Figure 7) [177]. Non-activated M0 macrophages are known to gain energy for ATP production mainly through oxidative phosphorylation, whereas M1 macrophages gain energy more by glycolysis, and M2 macrophages are characterized by moderate glycolytic activity and enhanced oxidative phosphorylation and fatty acid oxidation [178,179,180,181,182,183,184].

During M1 polarization, fatty acid synthesis is activated due to proinflammatory stimuli [185,186]. Fatty acid synthesis in these macrophages is carried out using substrates derived from other metabolic pathways, such as the truncated glycolytic pathway and the defective tricarboxylic acid (TCA) cycle, which lead to the accumulation of biosynthesis intermediate products [187,188], including citrate and succinate. These intermediates are used for both fatty acid synthesis [189,190] and proinflammatory mediators [187,189,191,192]. Indeed, carbon atoms derived from glucose at an increased rate of glycolysis in LPS-activated macrophages are preferentially incorporated into fatty acids and sterols [186,193].

In contrast to proinflammatory M1 macrophages, alternatively activated M2 macrophages use fatty acid oxidation [178,179,180,181]. In this case, fatty acid oxidation occurs in the mitochondria, as opposed to synthesis, which occurs in the cell cytoplasm.

Thus, the metabolic pathways in which fatty acids are involved and the phenotype of immune cells are closely linked, demonstrating different involvement in inflammation.

## 5. Short-Chain Fatty Acids

It is of interest to know that there is a metabolic and immune axis linking the lungs and the gut. These links are bidirectional, with the gut microbiome playing an important role in this interaction. The intestine is the principal site of localization for most of the commensal bacterial mass of the human microbiome [194,195]. This microbiome is metabolically active, being a source of several substances, such as short-chain fatty acids (SCFAs).

Short-chain fatty acids (SCFAs) are fatty acids with a straight or branched chain with less than six carbon atoms. The most common are acetate, propionate and butyrate, which are found in the colon in a molar ratio of approximately 57:22:21 [196,197]. SCFAs are produced by the intestinal microbiota as a result of anaerobic fermentation of dietary fiber. Important substrates for SCFAs formation are resistant starch, cellulose, and pectin [198]. In addition to carbohydrates, the formation of butyrate and propionate in the intestine also occurs as a result of the metabolism of organic acids and amino acids [199]. Protein fermentation can lead to the formation of branched-chain SCFAs, such as isobutyrate, 2-methylbutyrate, and isovalerate, derived from branched-chain amino acids (valine, isoleucine, and leucine) [200]. Metabolites of these amino acids may be associated with the development of insulin resistance [201].

SCFAs are found to a greater extent in the large intestine, where their concentration ranges from 70 to 130 mmol/kg, as well as in the bloodstream, but in much smaller amounts, amounting to approximately 0.1–5 μmol/L [197,202]. Most of the butyrate formed is used by colonocytes as an energy source, and these cells can obtain up to 60–70% of their energy from the oxidation of SCFAs [196,203]. Passing through the portal vein, propionate is metabolized by the liver, where it is used in gluconeogenesis [204,205], whereas most of the acetate enters the systemic bloodstream, where it is the most abundant SCFAs. The ratio of acetate, propionate, and butyrate in the portal vein is approximately 69:23:8 [196]. In plasma, the concentrations of acetate, propionate, and butyrate are approximately 25–250 μmol/L, 1.4–13.4 μmol/L, and 0.5–14.2 μmol/L, respectively [197,206]. It should be noted that plasma acetate may also have other origins, such as those associated with fatty acid oxidation and amino acid metabolism [207], ketogenesis in hepatocytes [208], or ethanol oxidation by microsomal cytochrome P450 enzymes [206,209].

The entry of SCFAs into the systemic bloodstream may be due both to passive diffusion and to the participation of special transporters, such as monocarboxylate transporter 1 (MCT1) and sodium-bound monocarboxylate transporter 1 (SMCT1) [198]. MCT1 has also been detected in cells of the immune system, including lymphocytes, monocytes, and neutrophils [210,211].

SCFAs are thought to realize their action through inhibition of histone deacetylase (HDAC) and through interaction with the G-protein-related receptors GPR43 and GPR41, also known as free fatty acid receptor (FFA)2 and FFA3, respectively [211,212,213,214]. In addition, the receptors for SCFAs are GPR109a (also known as HCA2) and olfactory receptor 78 (Olfr78) [215,216,217]. GPR43 is expressed in immune cells, including neutrophils, monocytes, and lymphocytes [211,212,213,218].

Butyrate, acetate, and propionate are considered to be histone deacetylases (HDAC) inhibitors, which are a class of enzymes that inhibit transcription through the removal of acetyl groups from chromatin [211,219]. Because of this, they are involved in the regulation of many cellular functions such as migration [211,220,221] and survival [211,222,223]. Butyrate, which is the strongest HDAC inhibitor [224,225], can cause macrophages to metabolically switch toward an anti-inflammatory M2 phenotype by inhibiting HDAC3 [226,227]. Another HDAC-related effect of butyrate is the inhibition of nitric oxide production (via iNOS) and lipopolysaccharide-induced proinflammatory cytokines (IL-6, IL-12) [227,228]. In addition, the anti-inflammatory effect of butyrate is associated with inhibition of the NF-kB signaling pathway as well as production by mononuclear cells and neutrophils of anti-inflammatory cytokines such as IL-10 [225,227].

Thus, SCFAs are believed to have anti-inflammatory and immunomodulatory effects [229]. SCFAs are involved in the regulation of differentiation, recruitment and activation of neutrophils, dendritic cells, macrophages and monocytes as well as T cells [206,215]. Butyrate reduces excessive airway infiltration by neutrophils through the GPCR-dependent receptor and by altering CXCL1 production [230].

SCFAs inhibit the maturation of monocytes, macrophages, and dendritic cells by altering their ability to capture antigens and reducing their ability to produce proinflammatory cytokines such as IL-12 and TNF-α [206,215,225]. Monocytes cultured in the presence of SCFAs show anti-inflammatory effects characterized by increased production of PGE2 [215,231].

The effect of SCFAs on cellular metabolism is of particular interest. Butyrate has been shown to promote memory potential in activated CD8+ T cells by influencing cellular metabolism [232].

A demonstration of the gut-lung connection is the detection of SCFAs in sputum [233]. In this connection, it is interesting to know that SCFAs can alter metabolic programming in LPS-exposed alveolar macrophages, which contributes to the maintenance of lung immunometabolic tone [234].

Interestingly, both butyrate and propionate restored and even improved the barrier function of the damaged airway epithelium, which may be mediated by increased expression of zonula occludens-1 (ZO-1) tight junction proteins [235]. Airway epithelial barrier dysfunction and dense contact disruption have been reported in asthma and in smoking and COPD [236,237]. In this regard, restoration of barrier function under the influence of SCFAs may have some clinical significance [235].

Other data suggest that the effect of SCFAs on lung cells can be not only anti-inflammatory but also pro-inflammatory, which depends on the type of cells studied and the concentration of SCFAs [238]. Interestingly, SCFAs in high concentrations caused significant inhibition of *P. aeruginosa* growth, which was enhanced at lower pH. At the same time, low concentrations of SCFAs resulted in enhanced bacterial growth [233].

These and other data suggest that SCFAs can act as pro- or anti-inflammatory molecules, depending on the cell type as well as on the conditions [215]. Research findings suggest that there is specificity in the immunomodulatory effects of butyrate, which may depend on the state of proliferation and activation in different cell types [239].

Given the link between the gut microbiome and lung function, there is increasing evidence of possible abnormalities in gut microflora in smoking and COPD [240,241]. In addition to smoking, chronic exposure to inhaled particulate matter, which is another important risk factor for COPD, in an experimental model in rats, causes gut dysbacteriosis and metabolic disorders [242].

It is believed that the most common bacteria in the intestine are representatives of *Bacteroidetes, Firmicutes*, which are mainly localized in the proximal colon [243,244,245]. They are involved in the production of SCFAs, and representatives of the *Bacteroidetes* type mainly produce acetate and propionate, while the *Firmicutes* type produces butyrate [196,246].

The available data suggest certain links between the intestinal and pulmonary microbiome [247]. Moreover, the diet may affect not only the gut microflora but also the respiratory tract microbiota [247,248]. Patients with chronic diseases show changes in the composition of the gut microflora with an increase in the number of harmful bacteria [249]. Interestingly, the proportion of *Bacteroidetes* is significantly reduced in COPD, which may contribute to the course of the disease [250,251]. In addition, the species diversity of the intestinal microflora and the number of *Bacteroides* decreases in the elderly [252,253].

**Figure 8:**
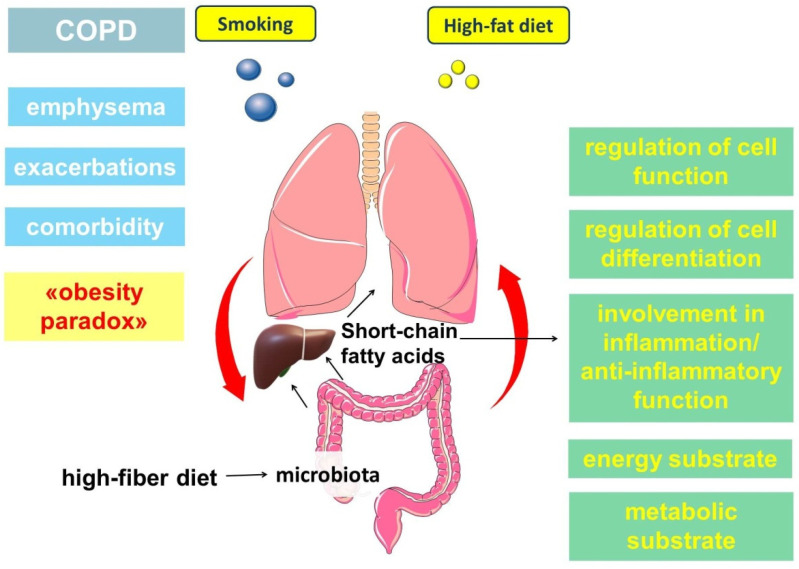
Schematic demonstrating the involvement of short-chain fatty acids in chronic obstructive pulmonary disease (COPD)-related biological processes.

It has also been shown that a high fat content in the diet leads to a decrease in the number of representatives of *Bacteroidetes* type [254,255,256]. Thus, the nature of the diet may influence not only the structure of the intestinal microbiota, but also the course of COPD through the regulation of many links of lipid metabolism (Figure 8).

## 6. The Importance of Nutrition in the Progression of COPD

The modern Western diet is considered an independent risk factor for many chronic noncommunicable diseases. Low levels of ω-3 PUFAs in the Western diet [257,258], may contribute to the development of some diseases, such as atherosclerosis, and may also be a factor associated with the prognosis of COPD. Several studies have demonstrated the association of ω-3 PUFAs levels in COPD with systemic inflammation and clinical outcomes [259,260,261]. In this case, adequate dietary intake of ω-3 PUFAs can be considered as a protective factor against the deterioration of lung function in smokers and the progression of COPD. Consumption of ω-3 PUFAs by COPD patients may be associated with weight gain and lower IL-6 levels compared with placebo [262].

PUFAs intake may also be associated with the severity of respiratory symptoms. A diet high in ω-3 PUFAs may help to reduce airway hypersensitivity and reduce the severity of exercise-induced bronchospasm [263,264,265]. It has also been shown that ω-3 PUFAs (eicosapentaenoic acid and docosapentaenoic acid) are associated with a reduced risk of non-specific bronchial hyperresponsiveness, whereas some ω-6 PUFAs, such as linoleic acid, dihomo-γ-linolenic acid, and arachidonic acid, are associated with an increased risk of non-specific bronchial hyperresponsiveness [265]. However, higher levels of eicosapentaenoic acid and docosahexaenoic acid were associated with a decreased likelihood of chronic cough [266]. These findings are of clinical interest given the frequent association of COPD with bronchial asthma and even the isolation of a separate phenotype, the so-called Asthma-COPD Overlap Syndrome (ACOS).

Despite these findings, there are still insufficient studies that can convincingly demonstrate the benefits of a diet rich in ω-3 PUFAs on the course and prognosis of COPD [141,267].

In addition to ω-3 PUFAs, nutritional support research in COPD patients has also focused on the role of sources of SCFAs. It has been shown that consumption of fruits, vegetables, oily fish, and whole-grain cereals may help protect against declining lung function in adults, especially in male smokers and patients with COPD [268]. High fiber intake has been inversely related to the incidence of COPD in both current and former male smokers [269]. At the same time, high fruit and vegetable intake in men was associated with decreased COPD incidence in both smokers and ex-smokers [270]. Interestingly, among women, reduced risk of COPD was associated with prolonged consumption of fruit rather than vegetables [271].

Weight loss and cachexia are important clinical characteristics of the adverse course of COPD [272]. Decreased body weight includes not only loss of adipose tissue, but also loss of muscle mass, which further impairs the physical activity and exercise capacity of patients. Decreased body mass index (BMI) values correlate well with predicted FEV1% and FEV1/FVC. Meanwhile, serum levels of ω-6 PUFAs metabolites such as linoleic acid, γ-linoleic acid, and arachidonic acid and ω-3 PUFAs metabolites such as eicosapentaenoic acid and docosahexaenoic acid show correlations with BMI and lung function [273]. PUFAs have also been shown to have a positive effect on exercise capacity in patients with COPD, which may be of clinical significance [274].

It has been suggested that overweight and obesity may be associated with changes in the composition of the intestinal microflora, including the ratio of *Bacteroidetes, Firmicutes* and others [275]. This can lead to changes in the production of SCFAs and their resulting effects. Plasma levels of butyrate/isobutyrate have been shown to be related to BMI [276]. An increase in BMI is accompanied by an increase in plasma butyrate/isobutyrate concentrations [276]. The results of low-fat/high-fiber diet experiments on a pig model showed increased production of SCFAs, especially butyrate by beneficial bacteria. Meanwhile, a high-fat/low-fiber diet for 7 weeks promoted increased bacterial development associated with negative health effects [277].

In another study, overweight and obese human volunteers were associated with a change in the ratio of individual SCFAs in favor of propionate [278]. Moreover, the total concentration of SCFAs in fecal samples was more than 20% higher in obese than in lean volunteers [278].

It has been shown that anorexia nervosa, demonstrates a decrease in intestinal microbial diversity associated with the production of SCFAs, primarily butyrate and propionate [279,280].

These findings are of particular interest given the paradoxical links between obesity and prognosis in COPD patients. Increased body weight and even obesity in these patients demonstrates better clinical outcomes. At the same time, decreased body weight in starvation, including anorexia nervosa, stimulates the development of emphysema.

It should be noted that there are associations between dietary precursors of SCFAs and the quantitative composition of plasma SCFAs. These relationships may be due to the fact that different sources of fermentable fiber can be differentially utilized by different composition of the gut microflora [276,281,282]. It has been shown that a diet high in fiber attenuated emphysema by suppressing airway inflammation. This could be due to the formation of SCFAs in the colon due to diet [251].

These data emphasize the importance of a comprehensive approach to the diet of COPD patients, taking into account the metabolic characteristics of individual food components [195].

## 7. Conclusions

The review of the literature suggests that COPD is characterized by the disruption of multiple lipid metabolic links (Table 1). Analyzing these data, one cannot ignore the heterogeneity of the disease itself. Many pathophysiological mechanisms of COPD heterogeneity are not yet clear, but the available data suggest that lipids may be involved in various links in the pathogenesis associated with COPD heterogeneity. Their complex links with emphysema, exacerbations, and comorbid diseases, such as the development of atherosclerosis, have been shown but not fully understood. There is no doubt that these links are multifaceted and include many links, the keys to understanding which may become more accessible with further study.

The links between fatty acid metabolism and the course of COPD are of great clinical interest and have been the subject of numerous studies. Their results demonstrate associations between decreased lung function and inflammation with dietary intake of ω-6 PUFAs [33,283,284]. Many studies have focused on assessing the clinical effectiveness of ω-3 PUFAs intake in COPD. However, these data cannot confirm with great certainty the existence of positive correlations between fatty acid intake and lung function as well as COPD progression and prognosis [285,286].

Lipid mediators associated with the resolution of inflammation are a promising new class of bioactive substances. They can be involved in many links of COPD pathogenesis and in doing so are considered as possible new targets for therapeutic action on inflammation.

Short-chain fatty acids are an important and interesting avenue for further scientific inquiry into the links between nutrition and COPD progression. Their better study may expand our understanding of the links between metabolism and inflammation and help improve nutritional support for COPD patients as an effective therapeutic intervention.

The available data suggest that some lipids, such as ceramides, are important markers of the course of COPD [287,288]. A better study of the role of fatty acids and their lipid mediators would allow integration of these data with clinical observations and an understanding of the natural history of COPD.

This review has shown that fatty acids and their metabolites exhibit multiple functions in inflammation. The understanding that fatty acids and lipid mediators may be involved in different phases of inflammation has greatly expanded the concepts of the complexity of their involvement in the pathogenesis of COPD.

## Figures and Tables

**Table 1:** Anti-inflammatory function of fatty acids and their metabolites in chronic obstructive pulmonary disease.

Lipids	Anti-Inflammatory Mechanisms	Changes in COPD	References
Long-chain polyunsaturated fatty acids	modulation of biophysical properties of plasma membranes (lipid ordering, fluidity, lipid rafts); regulation of membrane proteins function; gene expression (NF-kB, SREBP); substrate for synthesis of specialized pro-resolving mediators.	modification of the fatty acid composition of phospholipids of plasma membranes; lipid peroxidation; changes in the composition of free fatty acids; increased utilization of fatty acids.	[18,19,20,21,22,25,28,29,31,32,33,35,36,37,53,54,55,56,57]
Short-chain fatty acids	cell metabolism; cell differentiation; HDAC inhibition; modulation of mucosal inflammation; epithelial cell proliferation; junctional permeability.	changes in the composition of the intestinal microflora; eating disorders.	[204,209,210,211,212,213,214,221,222,223,224,225,229,230,231,232,233,236,237,238,239,240,241,244,245,246,247,248,249,254]
Specialized pro-resolving mediators	inhibition of neutrophil chemotaxis; inhibition of transendothelial and transepithelial migration of neutrophils; stimulation of phagocytosis and efferocytosis by macrophages; inhibition of cytokine production; influence on the proliferation of epithelial cells; participation in cholesterol homeostasis.	decreased production of pro-resolving mediators leads to: persistence of inflammation in the bronchi; development of emphysema; provides comorbid relationship with metabolic syndrome and atherosclerosis.	[9,10,60,67,68,69,70,71,72,73,75,76,77,78,79,80,81,82,83,84,85,86,87,88,89,90,91,92,93,94,95,98,99,100,101,103,105,106,107,108,109,110,111,112,113,114,115,116,117,118,119,120,121,122,129,130,131,132,133,134,135,136,137,138,141,142,143,150,151,153,154,155,156,157,158,159,162,163,164,165,167,168,170,171]

## Data Availability

Not applicable.

## References

[1] Chronic Obstructive Pulmonary Disease (COPD). https://www.who.int/news-room/fact-sheets/detail/chronic-obstructive-pulmonary-disease-.

[2] Mirza S., Benzo R. (2017). Chronic Obstructive Pulmonary Disease Phenotypes: Implications for Care. Mayo Clin. Proc..

[3] Kerkhof M., Voorham J., Dorinsky P., Cabrera C., Darken P., Kocks J.W.H., Sadatsafavi M., Sin D.D., Carter V., Price D.B. (2020). The Long-Term Burden of COPD Exacerbations During Maintenance Therapy and Lung Function Decline. Int. J. Chronic Obstr. Pulm. Dis..

[4] Viniol C., Vogelmeier C.F. (2018). Exacerbations of COPD. Eur. Respir. Rev..

[5] Le Rouzic O., Roche N., Cortot A.B., Tillie-Leblond I., Masure F., Perez T., Boucot I., Hamouti L., Ostinelli J., Pribil C. (2018). Defining the “Frequent Exacerbator” Phenotype in COPD: A Hypothesis-Free Approach. Chest.

[6] Motavkin P.A., Gelzer B.I. (1998). Clinical and Experimental Pathophysiology of the Lungs.

[7] Kotlyarov S., Kotlyarova A. (2021). Bioinformatic Analysis of ABCA1 Gene Expression in Smoking and Chronic Obstructive Pulmonary Disease. Membranes.

[8] Boukhenouna S., Wilson M.A., Bahmed K., Kosmider B. (2018). Reactive Oxygen Species in Chronic Obstructive Pulmonary Disease. Oxidative medicine and cellular longevity.

[9] Kotlyarov S., Kotlyarova A. (2021). Molecular Mechanisms of Lipid Metabolism Disorders in Infectious Exacerbations of Chronic Obstructive Pulmonary Disease. Int. J. Mol. Sci..

[10] Yang A., Wu Y., Yu G., Wang H. (2021). Role of specialized pro-resolving lipid mediators in pulmonary inflammation diseases: Mechanisms and development. Respir. Res..

[11] Fullerton J.N., Gilroy D.W. (2016). Resolution of inflammation: A new therapeutic frontier. Nat. Rev. Drug Discov..

[12] Newson J., Stables M., Karra E., Arce-Vargas F., Quezada S., Motwani M., Mack M., Yona S., Audzevich T., Gilroy D.W. (2014). Resolution of acute inflammation bridges the gap between innate and adaptive immunity. Blood.

[13] Wang B., Wu L., Chen J., Dong L., Chen C., Wen Z., Hu J., Fleming I., Wang D.W. (2021). Metabolism pathways of arachidonic acids: Mechanisms and potential therapeutic targets. Signal Transduct. Targe. Ther..

[14] Higgins A.J., Lees P. (1984). The acute inflammatory process, arachidonic acid metabolism and the mode of action of anti-inflammatory drugs. Equine Vet. J..

[15] Ibarguren M., López D.J., Escribá P.V. (2014). The effect of natural and synthetic fatty acids on membrane structure, microdomain organization, cellular functions and human health. Biochim. Biophys. Acta (BBA)-Biomembr..

[16] Caires R., Sierra-Valdez F.J., Millet J.R.M., Herwig J.D., Roan E., Vásquez V., Cordero-Morales J.F. (2017). Omega-3 Fatty Acids Modulate TRPV4 Function through Plasma Membrane Remodeling. Cell Rep..

[17] Zhu Y., Wen L., Wang S., Zhang K., Cui Y., Zhang C., Feng L., Yu F., Chen Y., Wang R. (2020). Omega-3 fatty acids improve flow-induced vasodilation by enhancing TRPV4 in arteries from diet-induced obese mice. Cardiovasc. Res..

[18] Weber J., Rajan S., Schremmer C., Chao Y.-K., Krasteva-Christ G., Kannler M., Yildirim A.Ö., Brosien M., Schredelseker J., Weissmann N. (2020). TRPV4 channels are essential for alveolar epithelial barrier function as protection from lung edema. JCI Insight.

[19] Pretorius E., du Plooy J.N., Soma P., Keyser I., Buys A.V. (2013). Smoking and fluidity of erythrocyte membranes: A high resolution scanning electron and atomic force microscopy investigation. Nitric Oxide.

[20] Hannan S.E., Harris J.O., Sheridan N.P., Patel J.M. (1989). Cigarette Smoke Alters Plasma Membrane Fluidity of Rat Alveolar Macrophages. Am. Rev. Respir. Dis..

[21] Padmavathi P., Reddy V.D., Maturu P., Varadacharyulu N. (2010). Smoking-Induced Alterations in Platelet Membrane Fluidity and Na^+^/K^+^-ATPase Activity in Chronic Cigarette Smokers. J. Atheroscler. Thromb..

[22] Rocha D.M., Caldas A.P., Oliveira L.L., Bressan J., Hermsdorff H.H. (2016). Saturated fatty acids trigger TLR4-mediated inflammatory response. Atherosclerosis.

[23] Xu X., Qi M.-Y., Liu S., Song X.-T., Zhang J.-N., Zhai Y.-F., Lu M.-H., Han H.-B., Lian Z.-X., Yao Y.-C. (2018). TLR4 overexpression enhances saturated fatty acid–induced inflammatory cytokine gene expression in sheep. Eur. J. Inflamm..

[24] Rogero M.M., Calder P.C. (2018). Obesity, Inflammation, Toll-Like Receptor 4 and Fatty Acids. Nutrients.

[25] Hoshino K., Takeuchi O., Kawai T., Sanjo H., Ogawa T., Takeda Y., Takeda K., Akira S. (1999). Cutting edge: Toll-like receptor 4 (TLR4)-deficient mice are hyporesponsive to lipopolysaccharide: Evidence for TLR4 as the Lps gene product. J. Immunol..

[26] Lee J.Y., Zhao L., Youn H.S., Weatherill A.R., Tapping R., Feng L., Lee W.H., Fitzgerald K.A., Hwang D.H. (2004). Saturated Fatty Acid Activates but Polyunsaturated Fatty Acid Inhibits Toll-like Receptor 2 Dimerized with Toll-like Receptor 6 or 1. J. Biol. Chem..

[27] Harb H., Irvine J., Amarasekera M., Hii C.S., Kesper D.A., Ma Y., D’Vaz N., Renz H., Potaczek D.P., Prescott S.L. (2017). The role of PKCζ in cord blood T-cell maturation towards Th1 cytokine profile and its epigenetic regulation by fish oil. Biosci. Rep..

[28] Acevedo N., Frumento P., Harb H., Alashkar Alhamwe B., Johansson C., Eick L., Alm J., Renz H., Scheynius A., Potaczek D.P. (2019). Histone Acetylation of Immune Regulatory Genes in Human Placenta in Association with Maternal Intake of Olive Oil and Fish Consumption. Int. J. Mol. Sci..

[29] van der Does A.M., Heijink M., Persson L.J., Kloos D.-P., Aanerud M., Bakke P., Taube C., Eagan T., Hiemstra P.S., Giera M. (2017). Disturbed fatty acid metabolism in airway secretions of patients with Chronic Obstructive Pulmonary Disease. Eur. Respir. J..

[30] Denisenko Y.K., Novgorodtseva T.P., Antonyuk M.V., Gvozdenko T.A., Zhukova N.V., Vitkina T.I., Gel’tser B.I. (2018). Pathogenesis of immune cell membrane abnormalities in comorbidity of chronic obstructive pulmonary disease and asthma. Pulmonologiya.

[31] Denisenko Y.K., Novgorodtseva T.P., Vitkina T.I., Antonyuk M.V., Bocharova N.V. (2018). The fatty acid composition of the mitochondrial membranes of platelets in chronic obstructive pulmonary disease. Klin. Med..

[32] van der Does A.M., Heijink M., Mayboroda O.A., Persson L.J., Aanerud M., Bakke P., Eagan T.M., Hiemstra P.S., Giera M. (2019). Dynamic differences in dietary polyunsaturated fatty acid metabolism in sputum of COPD patients and controls. Biochim. Biophys. Acta (BBA)-Mol. Cell Biol. Lipids.

[33] Gangopadhyay S., Vijayan V.K., Bansal S.K. (2012). Lipids of Erythrocyte Membranes of COPD Patients: A Quantitative and Qualitative Study. COPD J. Chronic Obstr. Pulm. Dis..

[34] Novgorodtseva T.P., Denisenko Y.K., Zhukova N.V., Antonyuk M.V., Knyshova V.V., Gvozdenko T.A. (2013). Modification of the fatty acid composition of the erythrocyte membrane in patients with chronic respiratory diseases. Lipids Health Dis..

[35] Novgorodtseva T.P., Denisenko Y.K., Antonyuk M.V., Zhukova N.V. (2013). Modification of fatty acid content of cell membranes of erythrocytes at chronic obstructive pulmonary disease. Bull. SB RAMS.

[36] Titz B., Luettich K., Leroy P., Boue S., Vuillaume G., Vihervaara T., Ekroos K., Martin F., Peitsch M.C., Hoeng J. (2016). Alterations in Serum Polyunsaturated Fatty Acids and Eicosanoids in Patients with Mild to Moderate Chronic Obstructive Pulmonary Disease (COPD). Int. J. Mol. Sci..

[37] Spiteller G. (2006). Peroxyl radicals: Inductors of neurodegenerative and other inflammatory diseases. Their origin and how they transform cholesterol, phospholipids, plasmalogens, polyunsaturated fatty acids, sugars, and proteins into deleterious products. Free Radic. Biol. Med..

[38] De Castro J., Hernández-Hernández A., Rodríguez M.C., Sardina J.L., Llanillo M., Sánchez-Yagüe J. (2007). Comparison of changes in erythrocyte and platelet phospholipid and fatty acid composition and protein oxidation in chronic obstructive pulmonary disease and asthma. Platelets.

[39] Jauregibeitia I., Portune K., Rica I., Tueros I., Velasco O., Grau G., Trebolazabala N., Castaño L., Larocca A.V., Ferreri C. (2020). Fatty Acid Profile of Mature Red Blood Cell Membranes and Dietary Intake as a New Approach to Characterize Children with Overweight and Obesity. Nutrients.

[40] Wada H., Goto H., Saitoh E., Ieki R., Okamura T., Ota T., Hagiwara S., Kodaka T., Yamamoto Y. (2005). Reduction in plasma free fatty acid in patients with chronic obstructive pulmonary disease. Am. J. Respir. Crit. Care Med..

[41] Agarwal A.R., Yin F., Cadenas E. (2014). Short-term cigarette smoke exposure leads to metabolic alterations in lung alveolar cells. Am. J. Respir. Cell Mol. Biol..

[42] Jiang Z., Knudsen N.H., Wang G., Qiu W., Naing Z.Z.C., Bai Y., Ai X., Lee C.-H., Zhou X. (2017). Genetic Control of Fatty Acid β-Oxidation in Chronic Obstructive Pulmonary Disease. Am. J. Respir. Cell Mol. Biol..

[43] Cornell K., Alam M., Lyden E., Wood L., LeVan T.D., Nordgren T.M., Bailey K., Hanson C. (2019). Saturated Fat Intake Is Associated with Lung Function in Individuals with Airflow Obstruction: Results from NHANES 2007–2012. Nutrients.

[44] Ceco E., Celli D., Weinberg S., Shigemura M., Welch L.C., Volpe L., Chandel N.S., Bharat A., Lecuona E., Sznajder J.I. (2021). Elevated CO(2) Levels Delay Skeletal Muscle Repair by Increasing Fatty Acid Oxidation. Front. Physiol..

[45] Cai B., Zhu Y., Ma Y.i., Xu Z., Zao Y.i., Wang J., Lin Y., Comer G.M. (2003). Effect of Supplementing a High-Fat, Low-Carbohydrate Enteral Formula in COPD Patients. Nutrition.

[46] Gong J., Zhao H., Liu T., Li L., Cheng E., Zhi S., Kong L., Yao H.-W., Li J. (2019). Cigarette Smoke Reduces Fatty Acid Catabolism, Leading to Apoptosis in Lung Endothelial Cells: Implication for Pathogenesis of COPD. Front. Physiol..

[47] Pullmannová P., Pavlíková L., Kováčik A., Sochorová M., Školová B., Slepička P., Maixner J., Zbytovská J., Vávrová K. (2017). Permeability and microstructure of model stratum corneum lipid membranes containing ceramides with long (C16) and very long (C24) acyl chains. Biophys. Chem..

[48] Sot J., Goñi F.M., Alonso A. (2005). Molecular associations and surface-active properties of short- and long-N-acyl chain ceramides. Biochim. Biophys. Acta.

[49] Sot J., Aranda F.J., Collado M.I., Goñi F.M., Alonso A. (2005). Different effects of long- and short-chain ceramides on the gel-fluid and lamellar-hexagonal transitions of phospholipids: A calorimetric, NMR, and X-ray diffraction study. Biophys. J..

[50] Stancevic B., Kolesnick R. (2010). Ceramide-rich platforms in transmembrane signaling. FEBS Lett..

[51] Goñi F.M., Contreras F.X., Montes L.R., Sot J., Alonso A. (2005). Biophysics (and sociology) of ceramides. Biochem. Soc. Symp..

[52] Balte P., Hoffman E.A., Oelsner E., Pistenmaa C.L., Michos E., Watson K., Laine A., Angelini E., Wysoczanski A., Stukovsky K.D.H. (2020). Associations of Plasma Omega-3 Fatty Acid Levels with Longitudinal Change in Percent Emphysema, Spirometry, and Chronic Lower Respiratory Disease Events: The Mesa Lung Study. C15. PREDICTING OUTCOMES IN COPD.

[53] Werz O., Gerstmeier J., Libreros S., De la Rosa X., Werner M., Norris P.C., Chiang N., Serhan C.N. (2018). Human macrophages differentially produce specific resolvin or leukotriene signals that depend on bacterial pathogenicity. Nat. Commun..

[54] Haeggström J.Z., Funk C.D. (2011). Lipoxygenase and leukotriene pathways: Biochemistry, biology, and roles in disease. Chem. Rev..

[55] Das U.N. (2021). “Cell Membrane Theory of Senescence” and the Role of Bioactive Lipids in Aging, and Aging Associated Diseases and Their Therapeutic Implications. Biomolecules.

[56] Das U.N. (2013). Arachidonic acid and lipoxin A4 as possible endogenous anti-diabetic molecules. Prostaglandins Leukot Essent Fatty Acids.

[57] Gundala N.K.V., Naidu V.G.M., Das U.N. (2017). Arachidonic acid and lipoxin A4 attenuate alloxan-induced cytotoxicity to RIN5F cells in vitro and type 1 diabetes mellitus in vivo. Biofactors.

[58] Gundala N.K.V., Naidu V.G.M., Das U.N. (2017). Arachidonic acid and lipoxinA4 attenuate streptozotocin-induced cytotoxicity to RIN5 F cells in vitro and type 1 and type 2 diabetes mellitus in vivo. Nutrition.

[59] Rutting S., Papanicolaou M., Xenaki D., Wood L.G., Mullin A.M., Hansbro P.M., Oliver B.G. (2018). Dietary ω-6 polyunsaturated fatty acid arachidonic acid increases inflammation, but inhibits ECM protein expression in COPD. Respir. Res..

[60] Balsinde J., Winstead M.V., Dennis E.A. (2002). Phospholipase A2 regulation of arachidonic acid mobilization. FEBS Lett..

[61] Basil M.C., Levy B.D. (2016). Specialized pro-resolving mediators: Endogenous regulators of infection and inflammation. Nat. Rev. Immunol..

[62] Leuti A., Maccarrone M., Chiurchiù V. (2019). Proresolving Lipid Mediators: Endogenous Modulators of Oxidative Stress. Oxidative medicine and cellular longevity.

[63] Serhan C.N., Levy B.D. (2018). Resolvins in inflammation: Emergence of the pro-resolving superfamily of mediators. J. Clin. Investig..

[64] Samuelsson B., Dahlén S.E., Lindgren J.A., Rouzer C.A., Serhan C.N. (1987). Leukotrienes and lipoxins: Structures, biosynthesis, and biological effects. Science.

[65] Malawista S.E., de Boisfleury Chevance A., van Damme J., Serhan C.N. (2008). Tonic inhibition of chemotaxis in human plasma. Proc. Natl. Acad. Sci. USA.

[66] Levy B.D., Clish C.B., Schmidt B., Gronert K., Serhan C.N. (2001). Lipid mediator class switching during acute inflammation: Signals in resolution. Nat. Immunol..

[67] Christman B.W., Christman J.W., Dworski R., Blair I.A., Prakash C. (1993). Prostaglandin E2 limits arachidonic acid availability and inhibits leukotriene B4 synthesis in rat alveolar macrophages by a nonphospholipase A2 mechanism. J. Immunol..

[68] Ye R.D., Boulay F., Wang J.M., Dahlgren C., Gerard C., Parmentier M., Serhan C.N., Murphy P.M. (2009). International Union of Basic and Clinical Pharmacology. LXXIII. Nomenclature for the formyl peptide receptor (FPR) family. Pharmacol. Rev..

[69] Duvall M.G., Bruggemann T.R., Levy B.D. (2017). Bronchoprotective mechanisms for specialized pro-resolving mediators in the resolution of lung inflammation. Mol. Aspects Med..

[70] Barnig C., Cernadas M., Dutile S., Liu X., Perrella M.A., Kazani S., Wechsler M.E., Israel E., Levy B.D. (2013). Lipoxin A4 regulates natural killer cell and type 2 innate lymphoid cell activation in asthma. Sci. Transl. Med..

[71] Bonnans C., Fukunaga K., Levy M.A., Levy B.D. (2006). Lipoxin A(4) regulates bronchial epithelial cell responses to acid injury. Am. J. Pathol..

[72] Chiang N., Serhan C.N., Dahlén S.E., Drazen J.M., Hay D.W., Rovati G.E., Shimizu T., Yokomizo T., Brink C. (2006). The lipoxin receptor ALX: Potent ligand-specific and stereoselective actions in vivo. Pharmacol. Rev..

[73] Fiore S., Maddox J.F., Perez H.D., Serhan C.N. (1994). Identification of a human cDNA encoding a functional high affinity lipoxin A4 receptor. J. Exp. Med..

[74] Hua J., Jin Y., Chen Y., Inomata T., Lee H., Chauhan S.K., Petasis N.A., Serhan C.N., Dana R. (2014). The resolvin D1 analogue controls maturation of dendritic cells and suppresses alloimmunity in corneal transplantation. Investig. Ophthalmol. Vis. Sci..

[75] Maddox J.F., Hachicha M., Takano T., Petasis N.A., Fokin V.V., Serhan C.N. (1997). Lipoxin A4 stable analogs are potent mimetics that stimulate human monocytes and THP-1 cells via a G-protein-linked lipoxin A4 receptor. J. Biol. Chem..

[76] Gastardelo T.S., Cunha B.R., Raposo L.S., Maniglia J.V., Cury P.M., Lisoni F.C., Tajara E.H., Oliani S.M. (2014). Inflammation and cancer: Role of annexin A1 and FPR2/ALX in proliferation and metastasis in human laryngeal squamous cell carcinoma. PLoS ONE.

[77] Serhan C.N., Maddox J.F., Petasis N.A., Akritopoulou-Zanze I., Papayianni A., Brady H.R., Colgan S.P., Madara J.L. (1995). Design of lipoxin A4 stable analogs that block transmigration and adhesion of human neutrophils. Biochemistry.

[78] Papayianni A., Serhan C.N., Brady H.R. (1996). Lipoxin A4 and B4 inhibit leukotriene-stimulated interactions of human neutrophils and endothelial cells. J. Immunol..

[79] Colgan S.P., Serhan C.N., Parkos C.A., Delp-Archer C., Madara J.L. (1993). Lipoxin A4 modulates transmigration of human neutrophils across intestinal epithelial monolayers. J. Clin. Investig..

[80] Maddox J.F., Serhan C.N. (1996). Lipoxin A4 and B4 are potent stimuli for human monocyte migration and adhesion: Selective inactivation by dehydrogenation and reduction. J. Exp. Med..

[81] Godson C., Mitchell S., Harvey K., Petasis N.A., Hogg N., Brady H.R. (2000). Cutting edge: Lipoxins rapidly stimulate nonphlogistic phagocytosis of apoptotic neutrophils by monocyte-derived macrophages. J. Immunol..

[82] McMahon B., Mitchell S., Brady H.R., Godson C. (2001). Lipoxins: Revelations on resolution. Trends Pharmacol. Sci..

[83] Higgins G., Ringholz F., Buchanan P., McNally P., Urbach V. (2015). Physiological impact of abnormal lipoxin A₄ production on cystic fibrosis airway epithelium and therapeutic potential. Biomed. Res. Int..

[84] Kenchegowda S., Bazan N.G., Bazan H.E. (2011). EGF stimulates lipoxin A4 synthesis and modulates repair in corneal epithelial cells through ERK and p38 activation. Investig. Ophthalmol. Vis. Sci..

[85] Higgins G., Buchanan P., Perriere M., Al-Alawi M., Costello R.W., Verriere V., McNally P., Harvey B.J., Urbach V. (2014). Activation of P2RY11 and ATP release by lipoxin A4 restores the airway surface liquid layer and epithelial repair in cystic fibrosis. Am. J. Respir. Cell Mol. Biol..

[86] József L., Zouki C., Petasis N.A., Serhan C.N., Filep J.G. (2002). Lipoxin A4 and aspirin-triggered 15-epi-lipoxin A4 inhibit peroxynitrite formation, NF-kappa B and AP-1 activation, and IL-8 gene expression in human leukocytes. Proc. Natl. Acad. Sci. USA.

[87] Bonnans C., Levy B.D. (2007). Lipid mediators as agonists for the resolution of acute lung inflammation and injury. Am. J. Respir. Cell Mol. Biol..

[88] Gewirtz A.T., McCormick B., Neish A.S., Petasis N.A., Gronert K., Serhan C.N., Madara J.L. (1998). Pathogen-induced chemokine secretion from model intestinal epithelium is inhibited by lipoxin A4 analogs. J. Clin. Investig..

[89] Verrière V., Grumbach Y., Chiron R., Urbach V. (2006). Lxa4 effect On intracellular Ca^2+^, Cl-secretion, tight junction formation and IL-8 production in normal and cf airway epithelium: 174. Pediatr. Pulmonol..

[90] Fierro I.M., Kutok J.L., Serhan C.N. (2002). Novel Lipid Mediator Regulators of Endothelial Cell Proliferation and Migration: Aspirin-Triggered-15R-Lipoxin A_4_ and Lipoxin A_4_. J. Pharmacol. Exp. Ther..

[91] El Kebir D., József L., Pan W., Wang L., Petasis N.A., Serhan C.N., Filep J.G. (2009). 15-epi-lipoxin A4 inhibits myeloperoxidase signaling and enhances resolution of acute lung injury. Am. J. Respir. Crit. Care Med..

[92] Balode L., Strazda G., Jurka N., Kopeika U., Kislina A., Beinare M., Bukovskis M., Taivans I. (2011). LSC 2011 Abstract: The role of lipoxin A4 in the chronic obstructive pulmonary disease. Eur. Respir. J..

[93] Balode L., Isajeva D., Kislina A., Isajevs S., Strazda G., Jurka N., Kopeika U., Bukovskis M., Taivans I. (2012). Chronic obstructive pulmonary disease is characterized with suppressed lipoxin A4 and increased lipoxin receptor expression in lungs. Eur. Respir. J..

[94] Fritscher L.G., Post M., Rodrigues M.T., Silverman F., Balter M., Chapman K.R., Zamel N. (2012). Profile of eicosanoids in breath condensate in asthma and COPD. J. Breath Res..

[95] Balode L., Isajevs S., Svirina D., Kopeika U., Strazda G., Taivans I. (2011). Lipoxin A4 receptor expression in smokers with and without COPD. Eur. Respir. J..

[96] Sha Y.-H., Hu Y.-W., Gao J.-J., Wang Y.-C., Ma X., Qiu Y.-R., Li S.-F., Zhao J.-Y., Huang C., Zhao J.-J. (2015). Lipoxin A4 promotes ABCA1 expression and cholesterol efflux through the LXRα signaling pathway in THP-1 macrophage-derived foam cells. Int. J. Clin. Exp. Pathol..

[97] Demetz E., Schroll A., Auer K., Heim C., Patsch J.R., Eller P., Theurl M., Theurl I., Theurl M., Seifert M. (2014). The Arachidonic Acid Metabolome Serves as a Conserved Regulator of Cholesterol Metabolism. Cell Metab..

[98] Yu D., Xu Z., Yin X., Zheng F., Lin X., Pan Q., Li H. (2015). Inverse Relationship between Serum Lipoxin A4 Level and the Risk of Metabolic Syndrome in a Middle-Aged Chinese Population. PLoS ONE.

[99] Ho K.J., Spite M., Owens C.D., Lancero H., Kroemer A.H., Pande R., Creager M.A., Serhan C.N., Conte M.S. (2010). Aspirin-triggered lipoxin and resolvin E1 modulate vascular smooth muscle phenotype and correlate with peripheral atherosclerosis. Am. J. Pathol..

[100] Libreros S., Shay A.E., Nshimiyimana R., Fichtner D., Martin M.J., Wourms N., Serhan C.N. (2021). A New E-Series Resolvin: RvE4 Stereochemistry and Function in Efferocytosis of Inflammation-Resolution. Front. Immunol..

[101] Krishnamoorthy S., Recchiuti A., Chiang N., Yacoubian S., Lee C.-H., Yang R., Petasis N.A., Serhan C.N. (2010). Resolvin D1 binds human phagocytes with evidence for proresolving receptors. Proc. Natl. Acad. Sci. UAS.

[102] Norling L.V., Dalli J., Flower R.J., Serhan C.N., Perretti M. (2012). Resolvin D1 limits polymorphonuclear leukocyte recruitment to inflammatory loci: Receptor-dependent actions. Arterioscler. Thromb. Vasc. Biol..

[103] Schmid M., Gemperle C., Rimann N., Hersberger M. (2016). Resolvin D1 Polarizes Primary Human Macrophages toward a Proresolution Phenotype through GPR32. J. Immunol..

[104] Chiang N., Fredman G., Bäckhed F., Oh S.F., Vickery T., Schmidt B.A., Serhan C.N. (2012). Infection regulates pro-resolving mediators that lower antibiotic requirements. Nature.

[105] Dalli J., Winkler J.W., Colas R.A., Arnardottir H., Cheng C.Y., Chiang N., Petasis N.A., Serhan C.N. (2013). Resolvin D3 and aspirin-triggered resolvin D3 are potent immunoresolvents. Chem. Biol..

[106] Krishnamoorthy S., Recchiuti A., Chiang N., Fredman G., Serhan C.N. (2012). Resolvin D1 receptor stereoselectivity and regulation of inflammation and proresolving microRNAs. Am. J. Pathol..

[107] Sun Y.P., Oh S.F., Uddin J., Yang R., Gotlinger K., Campbell E., Colgan S.P., Petasis N.A., Serhan C.N. (2007). Resolvin D1 and its aspirin-triggered 17R epimer. Stereochemical assignments, anti-inflammatory properties, and enzymatic inactivation. J. Biol. Chem..

[108] Kasuga K., Yang R., Porter T.F., Agrawal N., Petasis N.A., Irimia D., Toner M., Serhan C.N. (2008). Rapid Appearance of Resolvin Precursors in Inflammatory Exudates: Novel Mechanisms in Resolution. J. Immunol..

[109] Duffield J.S., Hong S., Vaidya V.S., Lu Y., Fredman G., Serhan C.N., Bonventre J.V. (2006). Resolvin D series and protectin D1 mitigate acute kidney injury. J. Immunol..

[110] Serhan C.N., Hong S., Gronert K., Colgan S.P., Devchand P.R., Mirick G., Moussignac R.-L. (2002). Resolvins a family of bioactive products of omega-3 fatty acid transformation circuits initiated by aspirin treatment that counter proinflammation signals. J. Exp. Med..

[111] Wang B., Gong X., Wan J.Y., Zhang L., Zhang Z., Li H.Z., Min S. (2011). Resolvin D1 protects mice from LPS-induced acute lung injury. Pulm. Pharmacol. Ther..

[112] Croasdell A., Thatcher T.H., Kottmann R.M., Colas R.A., Dalli J., Serhan C.N., Sime P.J., Phipps R.P. (2015). Resolvins attenuate inflammation and promote resolution in cigarette smoke-exposed human macrophages. Am. J. Physiol. Lung Cell. Mol. Physiol..

[113] Merched A.J., Ko K., Gotlinger K.H., Serhan C.N., Chan L. (2008). Atherosclerosis: Evidence for impairment of resolution of vascular inflammation governed by specific lipid mediators. FASEB J..

[114] Dalli J., Serhan C.N. (2012). Specific lipid mediator signatures of human phagocytes: Microparticles stimulate macrophage efferocytosis and pro-resolving mediators. Blood.

[115] Rogerio A.P., Haworth O., Croze R., Oh S.F., Uddin M., Carlo T., Pfeffer M.A., Priluck R., Serhan C.N., Levy B.D. (2012). Resolvin D1 and aspirin-triggered resolvin D1 promote resolution of allergic airways responses. J. Immunol..

[116] Abdulnour R.E., Sham H.P., Douda D.N., Colas R.A., Dalli J., Bai Y., Ai X., Serhan C.N., Levy B.D. (2016). Aspirin-triggered resolvin D1 is produced during self-resolving gram-negative bacterial pneumonia and regulates host immune responses for the resolution of lung inflammation. Mucosal Immunol..

[117] Hsiao H.M., Thatcher T.H., Colas R.A., Serhan C.N., Phipps R.P., Sime P.J. (2015). Resolvin D1 Reduces Emphysema and Chronic Inflammation. Am. J. Pathol..

[118] Hsiao H.M., Sapinoro R.E., Thatcher T.H., Croasdell A., Levy E.P., Fulton R.A., Olsen K.C., Pollock S.J., Serhan C.N., Phipps R.P. (2013). A novel anti-inflammatory and pro-resolving role for resolvin D1 in acute cigarette smoke-induced lung inflammation. PLoS ONE.

[119] Posso S.V., Quesnot N., Moraes J.A., Brito-Gitirana L., Kennedy-Feitosa E., Barroso M.V., Porto L.C., Lanzetti M., Valença S.S. (2018). AT-RVD1 repairs mouse lung after cigarette smoke-induced emphysema via downregulation of oxidative stress by NRF2/KEAP1 pathway. Int. Immunopharmacol..

[120] Xie W., Wang H., Liu Q., Li Y., Wang J., Yao S., Wu Q. (2016). ResolvinD1 reduces apoptosis and inflammation in primary human alveolar epithelial type 2 cells. Lab. Investig..

[121] Codagnone M., Cianci E., Lamolinara A., Mari V.C., Nespoli A., Isopi E., Mattoscio D., Arita M., Bragonzi A., Iezzi M. (2018). Resolvin D1 enhances the resolution of lung inflammation caused by long-term Pseudomonas aeruginosa infection. Mucosal Immunol..

[122] Pena K.B., Ramos C.O., Soares N.P., da Silva P.F., Bandeira A.C., Costa G.P., Cangussú S.D., Talvani A., Bezerra F.S. (2016). The administration of a high refined carbohydrate diet promoted an increase in pulmonary inflammation and oxidative stress in mice exposed to cigarette smoke. Int. J. Chronic Obstr. Pulm. Dis..

[123] Oh S.F., Dona M., Fredman G., Krishnamoorthy S., Irimia D., Serhan C.N. (2012). Resolvin E2 formation and impact in inflammation resolution. J. Immunol..

[124] Campbell E.L., Louis N.A., Tomassetti S.E., Canny G.O., Arita M., Serhan C.N., Colgan S.P. (2007). Resolvin E1 promotes mucosal surface clearance of neutrophils: A new paradigm for inflammatory resolution. FASEB J..

[125] Cash J.L., Bena S., Headland S.E., McArthur S., Brancaleone V., Perretti M. (2013). Chemerin15 inhibits neutrophil-mediated vascular inflammation and myocardial ischemia-reperfusion injury through ChemR23. EMBO Rep..

[126] Cash J.L., Hart R., Russ A., Dixon J.P., Colledge W.H., Doran J., Hendrick A.G., Carlton M.B., Greaves D.R. (2008). Synthetic chemerin-derived peptides suppress inflammation through ChemR23. J. Exp. Med..

[127] Du X.Y., Leung L.L. (2009). Proteolytic regulatory mechanism of chemerin bioactivity. Acta Biochim. Biophys. Sin..

[128] Herová M., Schmid M., Gemperle C., Hersberger M. (2015). ChemR23, the receptor for chemerin and resolvin E1, is expressed and functional on M1 but not on M2 macrophages. J. Immunol..

[129] Parolini S., Santoro A., Marcenaro E., Luini W., Massardi L., Facchetti F., Communi D., Parmentier M., Majorana A., Sironi M. (2007). The role of chemerin in the colocalization of NK and dendritic cell subsets into inflamed tissues. Blood.

[130] Samson M., Edinger A.L., Stordeur P., Rucker J., Verhasselt V., Sharron M., Govaerts C., Mollereau C., Vassart G., Doms R.W. (1998). ChemR23, a putative chemoattractant receptor, is expressed in monocyte-derived dendritic cells and macrophages and is a coreceptor for SIV and some primary HIV-1 strains. Eur. J. Immunol..

[131] Serhan C.N., Clish C.B., Brannon J., Colgan S.P., Chiang N., Gronert K. (2000). Novel functional sets of lipid-derived mediators with antiinflammatory actions generated from omega-3 fatty acids via cyclooxygenase 2-nonsteroidal antiinflammatory drugs and transcellular processing. J. Exp. Med..

[132] Tjonahen E., Oh S.F., Siegelman J., Elangovan S., Percarpio K.B., Hong S., Arita M., Serhan C.N. (2006). Resolvin E2: Identification and anti-inflammatory actions: Pivotal role of human 5-lipoxygenase in resolvin E series biosynthesis. Chem. Biol..

[133] Schwab J.M., Chiang N., Arita M., Serhan C.N. (2007). Resolvin E1 and protectin D1 activate inflammation-resolution programmes. Nature.

[134] Oh S.F., Pillai P.S., Recchiuti A., Yang R., Serhan C.N. (2011). Pro-resolving actions and stereoselective biosynthesis of 18S E-series resolvins in human leukocytes and murine inflammation. J. Clin. Investig..

[135] Arita M., Yoshida M., Hong S., Tjonahen E., Glickman J.N., Petasis N.A., Blumberg R.S., Serhan C.N. (2005). Resolvin E1, an endogenous lipid mediator derived from omega-3 eicosapentaenoic acid, protects against 2,4,6-trinitrobenzene sulfonic acid-induced colitis. Proc. Natl. Acad. Sci. USA.

[136] Seki H., Fukunaga K., Arita M., Arai H., Nakanishi H., Taguchi R., Miyasho T., Takamiya R., Asano K., Ishizaka A. (2010). The anti-inflammatory and proresolving mediator resolvin E1 protects mice from bacterial pneumonia and acute lung injury. J. Immunol..

[137] El Kebir D., Gjorstrup P., Filep J.G. (2012). Resolvin E1 promotes phagocytosis-induced neutrophil apoptosis and accelerates resolution of pulmonary inflammation. Proc. Natl. Acad. Sci. USA.

[138] Arita M., Ohira T., Sun Y.P., Elangovan S., Chiang N., Serhan C.N. (2007). Resolvin E1 selectively interacts with leukotriene B4 receptor BLT1 and ChemR23 to regulate inflammation. J. Immunol..

[139] Ohira T., Arita M., Omori K., Recchiuti A., Van Dyke T.E., Serhan C.N. (2010). Resolvin E1 receptor activation signals phosphorylation and phagocytosis. J. Biol. Chem..

[140] Pirault J., Bäck M. (2018). Lipoxin and Resolvin Receptors Transducing the Resolution of Inflammation in Cardiovascular Disease. Front. Pharmacol..

[141] Uppin V., Acharya P., Ravichandra Talahalli R. (2020). Modulatory Potentials of n-3 Polyunsaturated Fatty Acids in Inflammatory Diseases. Apolipoproteins, Triglycerides and Cholesterol.

[142] Spite M., Norling L.V., Summers L., Yang R., Cooper D., Petasis N.A., Flower R.J., Perretti M., Serhan C.N. (2009). Resolvin D2 is a potent regulator of leukocytes and controls microbial sepsis. Nature.

[143] Cash J.L., Norling L.V., Perretti M. (2014). Resolution of inflammation: Targeting GPCRs that interact with lipids and peptides. Drug Discov. Today.

[144] Isobe Y., Arita M., Matsueda S., Iwamoto R., Fujihara T., Nakanishi H., Taguchi R., Masuda K., Sasaki K., Urabe D. (2012). Identification and structure determination of novel anti-inflammatory mediator resolvin E3, 17,18-dihydroxyeicosapentaenoic acid. J Biol. Chem..

[145] Sato M., Aoki-Saito H., Fukuda H., Ikeda H., Koga Y., Yatomi M., Tsurumaki H., Maeno T., Saito T., Nakakura T. (2019). Resolvin E3 attenuates allergic airway inflammation via the interleukin-23-interleukin-17A pathway. FASEB J..

[146] Norris P.C., Libreros S., Serhan C.N. (2019). Resolution metabolomes activated by hypoxic environment. Sci. Adv..

[147] Hansen T.V., Vik A., Serhan C.N. (2019). The Protectin Family of Specialized Pro-resolving Mediators: Potent Immunoresolvents Enabling Innovative Approaches to Target Obesity and Diabetes. Front. Pharmacol..

[148] Tungen J.E., Aursnes M., Vik A., Ramon S., Colas R.A., Dalli J., Serhan C.N., Hansen T.V. (2014). Synthesis and anti-inflammatory and pro-resolving activities of 22-OH-PD1, a monohydroxylated metabolite of protectin D1. J. Nat. Prod..

[149] Mukherjee P.K., Marcheselli V.L., Serhan C.N., Bazan N.G. (2004). Neuroprotectin D1: A docosahexaenoic acid-derived docosatriene protects human retinal pigment epithelial cells from oxidative stress. Proc. Natl. Acad. Sci. USA.

[150] Bazan N.G. (2005). Neuroprotectin D1 (NPD1): A DHA-derived mediator that protects brain and retina against cell injury-induced oxidative stress. Brain Pathol..

[151] Serhan C.N., Fredman G., Yang R., Karamnov S., Belayev L.S., Bazan N.G., Zhu M., Winkler J.W., Petasis N.A. (2011). Novel proresolving aspirin-triggered DHA pathway. Chem. Biol..

[152] Bannenberg G.L., Chiang N., Ariel A., Arita M., Tjonahen E., Gotlinger K.H., Hong S., Serhan C.N. (2005). Molecular circuits of resolution: Formation and actions of resolvins and protectins. J. Immunol..

[153] Ariel A., Li P.L., Wang W., Tang W.X., Fredman G., Hong S., Gotlinger K.H., Serhan C.N. (2005). The docosatriene protectin D1 is produced by TH2 skewing and promotes human T cell apoptosis via lipid raft clustering. J. Biol. Chem..

[154] Ariel A., Fredman G., Sun Y.P., Kantarci A., Van Dyke T.E., Luster A.D., Serhan C.N. (2006). Apoptotic neutrophils and T cells sequester chemokines during immune response resolution through modulation of CCR5 expression. Nat. Immunol..

[155] Schett G., Neurath M.F. (2018). Resolution of chronic inflammatory disease: Universal and tissue-specific concepts. Nat. Commun..

[156] Wei J., Gronert K. (2017). The role of pro-resolving lipid mediators in ocular diseases. Mol. Aspects Med..

[157] Kraft J.D., Blomgran R., Lundgaard I., Quiding-Järbrink M., Bromberg J.S., Börgeson E. (2021). Specialized Pro-Resolving Mediators and the Lymphatic System. Int. J. Mol. Sci..

[158] Serhan C.N., Yang R., Martinod K., Kasuga K., Pillai P.S., Porter T.F., Oh S.F., Spite M. (2009). Maresins: Novel macrophage mediators with potent antiinflammatory and proresolving actions. J. Exp. Med..

[159] Rodriguez A.R., Spur B.W. (2012). Total synthesis of the macrophage derived anti-inflammatory lipid mediator Maresin 1. Tetrahedron Lett..

[160] Rodriguez A.R., Spur B.W. (2015). First total synthesis of the macrophage derived anti-inflammatory and pro-resolving lipid mediator Maresin 2. Tetrahedron Lett..

[161] Tang S., Wan M., Huang W., Stanton R.C., Xu Y. (2018). Maresins: Specialized Proresolving Lipid Mediators and Their Potential Role in Inflammatory-Related Diseases. Mediat. Inflamm..

[162] Li R., Wang Y., Ma Z., Ma M., Wang D., Xie G., Yin Y., Zhang P., Tao K. (2016). Maresin 1 Mitigates Inflammatory Response and Protects Mice from Sepsis. Mediat. Inflamm..

[163] Rodriguez A.R., Spur B.W. (2015). First total synthesis of pro-resolving and tissue-regenerative Maresin sulfido-conjugates. Tetrahedron Lett..

[164] Serhan C.N., Dalli J., Colas R.A., Winkler J.W., Chiang N. (2015). Protectins and maresins: New pro-resolving families of mediators in acute inflammation and resolution bioactive metabolome. Biochim. Biophys. Acta (BBA)-Mol. Cell Biol. Lipids.

[165] Li Y., Dalli J., Chiang N., Baron R.M., Quintana C., Serhan C.N. (2013). Plasticity of Leukocytic Exudates in Resolving Acute Inflammation Is Regulated by MicroRNA and Proresolving Mediators. Immunity.

[166] Serhan C.N., Chiang N., Dalli J. (2015). The resolution code of acute inflammation: Novel pro-resolving lipid mediators in resolution. Semin. Immunol..

[167] Serhan C.N., Dalli J., Karamnov S., Choi A., Park C.-K., Xu Z.-Z., Ji R.-R., Zhu M., Petasis N.A. (2012). Macrophage proresolving mediator maresin 1 stimulates tissue regeneration and controls pain. FASEB J..

[168] Sun Q., Wu Y., Zhao F., Wang J. (2017). Maresin 1 Ameliorates Lung Ischemia/Reperfusion Injury by Suppressing Oxidative Stress via Activation of the Nrf-2-Mediated HO-1 Signaling Pathway. Oxidative Med. Cell. Longev..

[169] Chiang N., Libreros S., Norris P.C., de la Rosa X., Serhan C.N. (2019). Maresin 1 activates LGR6 receptor promoting phagocyte immunoresolvent functions. J. Clin. Investig..

[170] Li Q.F., Hao H., Tu W.S., Guo N., Zhou X.Y. (2020). Maresins: Anti-inflammatory pro-resolving mediators with therapeutic potential. Eur. Rev. Med. Pharmacol. Sci..

[171] Qiu S., Li P., Zhao H., Li X. (2020). Maresin 1 alleviates dextran sulfate sodium-induced ulcerative colitis by regulating NRF2 and TLR4/NF-kB signaling pathway. Int. Immunopharmacol..

[172] Wang Q., Zhang H.-W., Mei H.-X., Ye Y., Xu H.-R., Xiang S.-Y., Yang Q., Zheng S.-X., Smith F.-G., Jin S.-W. (2020). MCTR1 enhances the resolution of lipopolysaccharide-induced lung injury through STAT6-mediated resident M2 alveolar macrophage polarization in mice. J. Cell. Mol. Med..

[173] Li H., Hao Y., Yang L.L., Wang X.Y., Li X.Y., Bhandari S., Han J., Liu Y.J., Gong Y.Q., Scott A. (2020). MCTR1 alleviates lipopolysaccharide-induced acute lung injury by protecting lung endothelial glycocalyx. J. Cell. Physiol..

[174] Ross E.A., Devitt A., Johnson J.R. (2021). Macrophages: The Good, the Bad, and the Gluttony. Front. Immunol..

[175] Saradna A., Do D.C., Kumar S., Fu Q.L., Gao P. (2018). Macrophage polarization and allergic asthma. Transl. Res..

[176] Abdelaziz M.H., Abdelwahab S.F., Wan J., Cai W., Huixuan W., Jianjun C., Kumar K.D., Vasudevan A., Sadek A., Su Z. (2020). Alternatively activated macrophages; a double-edged sword in allergic asthma. J. Transl. Med..

[177] Liu Y., Xu R., Gu H., Zhang E., Qu J., Cao W., Huang X., Yan H., He J., Cai Z. (2021). Metabolic reprogramming in macrophage responses. Biomark. Res..

[178] Remmerie A., Scott C.L. (2018). Macrophages and lipid metabolism. Cell. Immunol..

[179] O’Neill L.A.J., Kishton R.J., Rathmell J. (2016). A guide to immunometabolism for immunologists. Nat. Rev. Immunol..

[180] Odegaard J.I., Chawla A. (2011). Alternative Macrophage Activation and Metabolism. Annu. Rev. Pathol. Mech. Dis..

[181] Namgaladze D., Brüne B. (2016). Macrophage fatty acid oxidation and its roles in macrophage polarization and fatty acid-induced inflammation. Biochim. Biophys. Acta (BBA)-Mol. Cell Biol. Lipids.

[182] Lavin Y., Winter D., Blecher-Gonen R., David E., Keren-Shaul H., Merad M., Jung S., Amit I. (2014). Tissue-Resident Macrophage Enhancer Landscapes Are Shaped by the Local Microenvironment. Cell.

[183] Gautier E.L., Shay T., Miller J., Greter M., Jakubzick C., Ivanov S., Helft J., Chow A., Elpek K.G., Gordonov S. (2012). Gene-expression profiles and transcriptional regulatory pathways that underlie the identity and diversity of mouse tissue macrophages. Nat. Immunol..

[184] Kohyama M., Ise W., Edelson B.T., Wilker P.R., Hildner K., Mejia C., Frazier W.A., Murphy T.L., Murphy K.M. (2009). Role for Spi-C in the development of red pulp macrophages and splenic iron homeostasis. Nature.

[185] Posokhova E., Khoshchenko O., Chasovskikh M., Pivovarova E., Dushkin M. (2008). Lipid synthesis in macrophages during inflammation in vivo: Effect of agonists of peroxisome proliferator activated receptors α and γ and of retinoid X receptors. Biochemistry.

[186] Feingold K.R., Shigenaga J.K., Kazemi M.R., McDonald C.M., Patzek S.M., Cross A.S., Moser A., Grunfeld C. (2012). Mechanisms of triglyceride accumulation in activated macrophages. J. Leukoc. Biol..

[187] Michaeloudes C., Bhavsar P.K., Mumby S., Xu B., Hui C.K.M., Chung K.F., Adcock I.M. (2020). Role of Metabolic Reprogramming in Pulmonary Innate Immunity and Its Impact on Lung Diseases. J. Innate Immun..

[188] Everts B., Amiel E., Huang S.C., Smith A.M., Chang C.H., Lam W.Y., Redmann V., Freitas T.C., Blagih J., van der Windt G.J. (2014). TLR-driven early glycolytic reprogramming via the kinases TBK1-IKKɛ supports the anabolic demands of dendritic cell activation. Nat. Immunol..

[189] Jha A.K., Huang S.C., Sergushichev A., Lampropoulou V., Ivanova Y., Loginicheva E., Chmielewski K., Stewart K.M., Ashall J., Everts B. (2015). Network integration of parallel metabolic and transcriptional data reveals metabolic modules that regulate macrophage polarization. Immunity.

[190] Wei X., Song H., Yin L., Rizzo M.G., Sidhu R., Covey D.F., Ory D.S., Semenkovich C.F. (2016). Fatty acid synthesis configures the plasma membrane for inflammation in diabetes. Nature.

[191] Van den Bossche J., O’Neill L.A., Menon D. (2017). Macrophage Immunometabolism: Where Are We (Going)?. Trends Immunol..

[192] Tannahill G.M., Curtis A.M., Adamik J., Palsson-McDermott E.M., McGettrick A.F., Goel G., Frezza C., Bernard N.J., Kelly B., Foley N.H. (2013). Succinate is an inflammatory signal that induces IL-1β through HIF-1α. Nature.

[193] Diskin C., Pålsson-McDermott E.M. (2018). Metabolic Modulation in Macrophage Effector Function. Front. Immunol..

[194] Wang B., Yao M., Lv L., Ling Z., Li L. (2017). The Human Microbiota in Health and Disease. Engineering.

[195] Vaughan A., Frazer Z.A., Hansbro P.M., Yang I.A. (2019). COPD and the gut-lung axis: The therapeutic potential of fibre. J. Thorac. Dis..

[196] den Besten G., van Eunen K., Groen A.K., Venema K., Reijngoud D.-J., Bakker B.M. (2013). The role of short-chain fatty acids in the interplay between diet, gut microbiota, and host energy metabolism. J. Lipid Res..

[197] Cummings J.H., Pomare E.W., Branch W.J., Naylor C.P., Macfarlane G.T. (1987). Short chain fatty acids in human large intestine, portal, hepatic and venous blood. Gut.

[198] Parada Venegas D., De la Fuente M.K., Landskron G., González M.J., Quera R., Dijkstra G., Harmsen H.J.M., Faber K.N., Hermoso M.A. (2019). Short Chain Fatty Acids (SCFAs)-Mediated Gut Epithelial and Immune Regulation and Its Relevance for Inflammatory Bowel Diseases. Front. Immunol..

[199] Louis P., Flint H.J. (2017). Formation of propionate and butyrate by the human colonic microbiota. Environ. Microbiol..

[200] Smith E.A., Macfarlane G.T. (1997). Dissimilatory amino Acid metabolism in human colonic bacteria. Anaerobe.

[201] Newgard C.B., An J., Bain J.R., Muehlbauer M.J., Stevens R.D., Lien L.F., Haqq A.M., Shah S.H., Arlotto M., Slentz C.A. (2009). A branched-chain amino acid-related metabolic signature that differentiates obese and lean humans and contributes to insulin resistance. Cell Metab..

[202] Mortensen P.B., Clausen M.R. (1996). Short-chain fatty acids in the human colon: Relation to gastrointestinal health and disease. Scand. J. Gastroenterol. Suppl..

[203] Roediger W.E. (1982). Utilization of nutrients by isolated epithelial cells of the rat colon. Gastroenterology.

[204] Roy C.C., Kien C.L., Bouthillier L., Levy E. (2006). Short-chain fatty acids: Ready for prime time?. Nutr. Clin. Pract..

[205] Wiltrout D.W., Satter L.D. (1972). Contribution of propionate to glucose synthesis in the lactating and nonlactating cow. J. Dairy Sci..

[206] Dalile B., Van Oudenhove L., Vervliet B., Verbeke K. (2019). The role of short-chain fatty acids in microbiota–gut–brain communication. Nat. Rev. Gastroenterol. Hepatol..

[207] Layden B.T., Angueira A.R., Brodsky M., Durai V., Lowe W.L. (2013). Short chain fatty acids and their receptors: New metabolic targets. Transl. Res..

[208] Yamashita H., Kaneyuki T., Tagawa K. (2001). Production of acetate in the liver and its utilization in peripheral tissues. Biochim. Biophys. Acta.

[209] Bell-Parikh L.C., Guengerich F.P. (1999). Kinetics of cytochrome P450 2E1-catalyzed oxidation of ethanol to acetic acid via acetaldehyde. J. Biol. Chem..

[210] Merezhinskaya N., Ogunwuyi S.A., Mullick F.G., Fishbein W.N. (2004). Presence and localization of three lactic acid transporters (MCT1, -2, and -4) in separated human granulocytes, lymphocytes, and monocytes. J. Histochem. Cytochem..

[211] Sturm E.M., Knuplez E., Marsche G. (2021). Role of Short Chain Fatty Acids and Apolipoproteins in the Regulation of Eosinophilia-Associated Diseases. Int. J. Mol. Sci..

[212] Le Poul E., Loison C., Struyf S., Springael J.-Y., Lannoy V., Decobecq M.-E., Brezillon S., Dupriez V., Vassart G., Van Damme J. (2003). Functional characterization of human receptors for short chain fatty acids and their role in polymorphonuclear cell activation. J. Biol. Chem..

[213] Nilsson N.E., Kotarsky K., Owman C., Olde B. (2003). Identification of a free fatty acid receptor, FFA2R, expressed on leukocytes and activated by short-chain fatty acids. Biochem. Biophys. Res. Commun..

[214] Ulven T. (2012). Short-chain free fatty acid receptors FFA2/GPR43 and FFA3/GPR41 as new potential therapeutic targets. Front. Endocrinol..

[215] Corrêa-Oliveira R., Fachi J.L., Vieira A., Sato F.T., Vinolo M.A.R. (2016). Regulation of immune cell function by short-chain fatty acids. Clin. Transl. Immunol..

[216] Pluznick J. (2014). A novel SCFA receptor, the microbiota, and blood pressure regulation. Gut Microbes.

[217] Thangaraju M., Cresci G.A., Liu K., Ananth S., Gnanaprakasam J.P., Browning D.D., Mellinger J.D., Smith S.B., Digby G.J., Lambert N.A. (2009). GPR109A is a G-protein-coupled receptor for the bacterial fermentation product butyrate and functions as a tumor suppressor in colon. Cancer Res..

[218] Maslowski K.M., Vieira A.T., Ng A., Kranich J., Sierro F., Yu D., Schilter H.C., Rolph M.S., Mackay F., Artis D. (2009). Regulation of inflammatory responses by gut microbiota and chemoattractant receptor GPR43. Nature.

[219] Bolden J.E., Peart M.J., Johnstone R.W. (2006). Anticancer activities of histone deacetylase inhibitors. Nature Rev. Drug Discov..

[220] Lin K.T., Wang Y.W., Chen C.T., Ho C.M., Su W.H., Jou Y.S. (2012). HDAC inhibitors augmented cell migration and metastasis through induction of PKCs leading to identification of low toxicity modalities for combination cancer therapy. Clin. Cancer Res..

[221] Xu Z., Tao J., Chen P., Chen L., Sharma S., Wang G., Dong Q. (2018). Sodium butyrate inhibits colorectal cancer cell migration by downregulating Bmi-1 through enhanced miR-200c expression. Mol. Nutr. Food Res..

[222] Kankaanranta H., Janka-Junttila M., Ilmarinen-Salo P., Ito K., Jalonen U., Ito M., Adcock I.M., Moilanen E., Zhang X. (2010). Histone deacetylase inhibitors induce apoptosis in human eosinophils and neutrophils. J. Inflamm..

[223] Aoyama M., Kotani J., Usami M. (2010). Butyrate and propionate induced activated or non-activated neutrophil apoptosis via HDAC inhibitor activity but without activating GPR-41/GPR-43 pathways. Nutrition.

[224] Park J., Kim M., Kang S.G., Jannasch A.H., Cooper B., Patterson J., Kim C.H. (2015). Short-chain fatty acids induce both effector and regulatory T cells by suppression of histone deacetylases and regulation of the mTOR–S6K pathway. Mucosal Immunol..

[225] Chang P.V., Hao L., Offermanns S., Medzhitov R. (2014). The microbial metabolite butyrate regulates intestinal macrophage function via histone deacetylase inhibition. Proc. Natl. Acad. Sci. USA.

[226] Zhang Z., Tang H., Chen P., Xie H., Tao Y. (2019). Demystifying the manipulation of host immunity, metabolism, and extraintestinal tumors by the gut microbiome. Signal Transduct. Target. Ther..

[227] Jardou M., Lawson R. (2021). Supportive therapy during COVID-19: The proposed mechanism of short-chain fatty acids to prevent cytokine storm and multi-organ failure. Med. Hypotheses.

[228] He J., Zhang P., Shen L., Niu L., Tan Y., Chen L., Zhao Y., Bai L., Hao X., Li X. (2020). Short-Chain Fatty Acids and Their Association with Signalling Pathways in Inflammation, Glucose and Lipid Metabolism. Int. J. Mol. Sci..

[229] Yoon H.J., Park M.K., Lee H., Park T.S., Park D.W., Moon J.-Y., Kim T.H., Sohn J.W., Kim S.-H., Yoon H.-R. (2020). Effects of respiratory short-chain fatty acids on bronchial inflammation in asthma. World Allergy Organ. J..

[230] Trompette A., Gollwitzer E.S., Pattaroni C., Lopez-Mejia I.C., Riva E., Pernot J., Ubags N., Fajas L., Nicod L.P., Marsland B.J. (2018). Dietary Fiber Confers Protection against Flu by Shaping Ly6c(-) Patrolling Monocyte Hematopoiesis and CD8(+) T Cell Metabolism. Immunity.

[231] Cox M.A., Jackson J., Stanton M., Rojas-Triana A., Bober L., Laverty M., Yang X., Zhu F., Liu J., Wang S. (2009). Short-chain fatty acids act as antiinflammatory mediators by regulating prostaglandin E(2) and cytokines. World J. Gastroenterol..

[232] Bachem A., Makhlouf C., Binger K.J., de Souza D.P., Tull D., Hochheiser K., Whitney P.G., Fernandez-Ruiz D., Dähling S., Kastenmüller W. (2019). Microbiota-Derived Short-Chain Fatty Acids Promote the Memory Potential of Antigen-Activated CD8+ T Cells. Immunity.

[233] Ghorbani P., Santhakumar P., Hu Q., Djiadeu P., Wolever T.M., Palaniyar N., Grasemann H. (2015). Short-chain fatty acids affect cystic fibrosis airway inflammation and bacterial growth. Eur. Respir. J..

[234] Liu Q., Tian X., Maruyama D., Arjomandi M., Prakash A. (2021). Lung immune tone via gut-lung axis: Gut-derived LPS and short-chain fatty acids’ immunometabolic regulation of lung IL-1β, FFAR2, and FFAR3 expression. Am. J. Physiol.-Lung Cell. Mol. Physiol..

[235] Richards L.B., Li M., Folkerts G., Henricks P.A.J., Garssen J., van Esch B.C.A.M. (2020). Butyrate and Propionate Restore the Cytokine and House Dust Mite Compromised Barrier Function of Human Bronchial Airway Epithelial Cells. Int. J. Mol. Sci..

[236] Schamberger A.C., Mise N., Jia J., Genoyer E., Yildirim A., Meiners S., Eickelberg O. (2014). Cigarette smoke-induced disruption of bronchial epithelial tight junctions is prevented by transforming growth factor-β. Am. J. Respir. Cell Mol. Biol..

[237] Tatsuta M., Kan-o K., Ishii Y., Yamamoto N., Ogawa T., Fukuyama S., Ogawa A., Fujita A., Nakanishi Y., Matsumoto K. (2019). Effects of cigarette smoke on barrier function and tight junction proteins in the bronchial epithelium: Protective role of cathelicidin LL-37. Respir. Res..

[238] Rutting S., Xenaki D., Malouf M., Horvat J.C., Wood L.G., Hansbro P.M., Oliver B.G. (2019). Short-chain fatty acids increase TNFα-induced inflammation in primary human lung mesenchymal cells through the activation of p38 MAPK. Am. J. Physiol. Cell. Mol. Physiol..

[239] Bailón E., Cueto-Sola M., Utrilla P., Rodríguez-Cabezas M.E., Garrido-Mesa N., Zarzuelo A., Xaus J., Gálvez J., Comalada M. (2010). Butyrate in vitro immune-modulatory effects might be mediated through a proliferation-related induction of apoptosis. Immunobiology.

[240] Sze M.A., Hogg J.C., Sin D.D. (2014). Bacterial microbiome of lungs in COPD. Int. J. Chronic Obstr. Pulm. Dis..

[241] Biedermann L., Zeitz J., Mwinyi J., Sutter-Minder E., Rehman A., Ott S.J., Steurer-Stey C., Frei A., Frei P., Scharl M. (2013). Smoking cessation induces profound changes in the composition of the intestinal microbiota in humans. PLoS ONE.

[242] Li N., Yang Z., Liao B., Pan T., Pu J., Hao B., Fu Z., Cao W., Zhou Y., He F. (2020). Chronic exposure to ambient particulate matter induces gut microbial dysbiosis in a rat COPD model. Respir. Res..

[243] Reiss A., Jacobi M., Rusch K., Schwiertz A. (2016). Association of dietary type with fecal microbiota and short chain fatty acids in vegans and omnivores. J. Int. Soc. Microbiota.

[244] Eckburg P.B., Bik E.M., Bernstein C.N., Purdom E., Dethlefsen L., Sargent M., Gill S.R., Nelson K.E., Relman D.A. (2005). Diversity of the human intestinal microbial flora. Science.

[245] Alonso V.R., Guarner F. (2013). Intestinal microbiota composition in adults. Probiotic Bacteria and Their Effect on Human Health and Well-Being.

[246] Macfarlane S., Macfarlane G.T. (2003). Regulation of short-chain fatty acid production. Proc. Nutr. Soc..

[247] Madan J.C., Koestler D.C., Stanton B.A., Davidson L., Moulton L.A., Housman M.L., Moore J.H., Guill M.F., Morrison H.G., Sogin M.L. (2012). Serial Analysis of the Gut and Respiratory Microbiome in Cystic Fibrosis in Infancy: Interaction between Intestinal and Respiratory Tracts and Impact of Nutritional Exposures. mBio.

[248] Trompette A., Gollwitzer E.S., Yadava K., Sichelstiel A.K., Sprenger N., Ngom-Bru C., Blanchard C., Junt T., Nicod L.P., Harris N.L. (2014). Gut microbiota metabolism of dietary fiber influences allergic airway disease and hematopoiesis. Nat. Med..

[249] Marsland B.J., Trompette A., Gollwitzer E.S. (2015). The Gut-Lung Axis in Respiratory Disease. Ann. Am. Thorac. Soc..

[250] Sze M.A., Dimitriu P.A., Hayashi S., Elliott W.M., McDonough J.E., Gosselink J.V., Cooper J., Sin D.D., Mohn W.W., Hogg J.C. (2012). The lung tissue microbiome in chronic obstructive pulmonary disease. Am. J. Respir. Crit. Care Med..

[251] Jang Y.O., Kim O.-H., Kim S.J., Lee S.H., Yun S., Lim S.E., Yoo H.J., Shin Y., Lee S.W. (2021). High-fiber diets attenuate emphysema development via modulation of gut microbiota and metabolism. Sci. Rep..

[252] Woodmansey E.J. (2007). Intestinal bacteria and ageing. J. Appl. Microbiol..

[253] Woodmansey E.J., McMurdo M.E., Macfarlane G.T., Macfarlane S. (2004). Comparison of compositions and metabolic activities of fecal microbiotas in young adults and in antibiotic-treated and non-antibiotic-treated elderly subjects. Appl. Environ. Microbiol..

[254] Murphy E.F., Cotter P.D., Healy S., Marques T.M., O’Sullivan O., Fouhy F., Clarke S.F., O’Toole P.W., Quigley E.M., Stanton C. (2010). Composition and energy harvesting capacity of the gut microbiota: Relationship to diet, obesity and time in mouse models. Gut.

[255] Hildebrandt M.A., Hoffmann C., Sherrill–Mix S.A., Keilbaugh S.A., Hamady M., Chen Y.Y., Knight R., Ahima R.S., Bushman F., Wu G.D. (2009). High-fat diet determines the composition of the murine gut microbiome independently of obesity. Gastroenterology.

[256] Senghor B., Sokhna C., Ruimy R., Lagier J.-C. (2018). Gut microbiota diversity according to dietary habits and geographical provenance. Hum. Microbiome J..

[257] Simopoulos A.P. (2009). Omega-6/omega-3 essential fatty acids: Biological effects. World Rev. Nutr. Diet.

[258] Simopoulos A.P. (2011). Importance of the omega-6/omega-3 balance in health and disease: Evolutionary aspects of diet. World Rev. Nutr. Diet.

[259] Wood L.G., Scott H.A., Garg M.L., Gibson P.G. (2009). Innate immune mechanisms linking non-esterified fatty acids and respiratory disease. Prog. Lipid Res..

[260] Shahar E., Boland L.L., Folsom A.R., Tockman M.S., McGovern P.G., Eckfeldt J.H. (1999). Docosahexaenoic acid and smoking-related chronic obstructive pulmonary disease. The Atherosclerosis Risk in Communities Study Investigators. Am. J. Respir. Crit. Care Med..

[261] Hirayama F., Lee A.H., Binns C.W., Hiramatsu N., Mori M., Nishimura K. (2010). Dietary intake of isoflavones and polyunsaturated fatty acids associated with lung function, breathlessness and the prevalence of chronic obstructive pulmonary disease: Possible protective effect of traditional Japanese diet. Mol. Nutr. Food Res..

[262] Yu H., Su X., Lei T., Zhang C., Zhang M., Wang Y., Zhu L., Liu J. (2021). Effect of Omega-3 Fatty Acids on Chronic Obstructive Pulmonary Disease: A Systematic Review and Meta-Analysis of Randomized Controlled Trials. Int. J. Chronic Obstr. Pulm. Dis..

[263] Mickleborough T.D., Lindley M.R., Ionescu A.A., Fly A.D. (2006). Protective effect of fish oil supplementation on exercise-induced bronchoconstriction in asthma. Chest.

[264] Mickleborough T.D., Murray R.L., Ionescu A.A., Lindley M.R. (2003). Fish oil supplementation reduces severity of exercise-induced bronchoconstriction in elite athletes. Am. J. Respir. Crit. Care Med..

[265] Adams S., Lopata A.L., Smuts C.M., Baatjies R., Jeebhay M.F. (2018). Relationship between Serum Omega-3 Fatty Acid and Asthma Endpoints. Int. J. Environ. Res. Public Health.

[266] Lemoine S.C.M., Brigham E.P., Woo H., Hanson C.K., McCormack M.C., Koch A., Putcha N., Hansel N.N. (2019). Omega-3 fatty acid intake and prevalent respiratory symptoms among U.S. adults with COPD. BMC Pulm. Med..

[267] Shahar E., Folsom A.R., Melnick S.L., Tockman M.S., Comstock G.W., Gennaro V., Higgins M.W., Sorlie P.D., Ko W.-J., Szklo M. (1994). Dietary n-3 Polyunsaturated Fatty Acids and Smoking-Related Chronic Obstructive Pulmonary Disease. New Engl. J. Med..

[268] Shaheen S.O., Jameson K.A., Syddall H.E., Aihie Sayer A., Dennison E.M., Cooper C., Robinson S.M., Group T.H.C.S. (2010). The relationship of dietary patterns with adult lung function and COPD. Eur. Respir. J..

[269] Kaluza J., Harris H., Wallin A., Linden A., Wolk A. (2018). Dietary Fiber Intake and Risk of Chronic Obstructive Pulmonary Disease: A Prospective Cohort Study of Men. Epidemiology.

[270] Kaluza J., Larsson S.C., Orsini N., Linden A., Wolk A. (2017). Fruit and vegetable consumption and risk of COPD: A prospective cohort study of men. Thorax.

[271] Kaluza J., Harris H.R., Linden A., Wolk A. (2018). Long-term consumption of fruits and vegetables and risk of chronic obstructive pulmonary disease: A prospective cohort study of women. Int. J. Epidemiol..

[272] Kotlyarov S., Kotlyarova A. (2021). Atherosclerosis as a risk factor in the prognosis of the survival of patients with COPD. Eur. Heart J. Acute Cardiovasc. Care.

[273] Xue M., Cai C., Guan L., Xu Y., Lin J., Zeng Y., Hu H., Chen R., Wang H., Zhou L. (2020). Exploration of n-6 and n-3 Polyunsaturated Fatty Acids Metabolites Associated with Nutritional Levels in Patients with Severe Stable Chronic Obstructive Pulmonary Disease. Int. J. Chronic Obstr. Pulm. Dis..

[274] Broekhuizen R., Wouters E.F., Creutzberg E.C., Weling-Scheepers C.A., Schols A.M. (2005). Polyunsaturated fatty acids improve exercise capacity in chronic obstructive pulmonary disease. Thorax.

[275] Angelakis E., Armougom F., Million M., Raoult D. (2012). The relationship between gut microbiota and weight gain in humans. Future Microbiol..

[276] Wang Y., Wang H., Howard A.G., Meyer K.A., Tsilimigras M.C.B., Avery C.L., Sha W., Sun S., Zhang J., Su C. (2020). Circulating Short-Chain Fatty Acids Are Positively Associated with Adiposity Measures in Chinese Adults. Nutrients.

[277] Heinritz S.N., Weiss E., Eklund M., Aumiller T., Louis S., Rings A., Messner S., Camarinha-Silva A., Seifert J., Bischoff S.C. (2016). Intestinal Microbiota and Microbial Metabolites Are Changed in a Pig Model Fed a High-Fat/Low-Fiber or a Low-Fat/High-Fiber Diet. PLoS ONE.

[278] Schwiertz A., Taras D., Schäfer K., Beijer S., Bos N.A., Donus C., Hardt P.D. (2010). Microbiota and SCFA in lean and overweight healthy subjects. Obesity.

[279] Reed K.K., Abbaspour A., Bulik C.M., Carroll I.M. (2021). The intestinal microbiota and anorexia nervosa: Cause or consequence of nutrient deprivation. Curr. Opin. Endocr. Metab. Res..

[280] Mack I., Cuntz U., Grämer C., Niedermaier S., Pohl C., Schwiertz A., Zimmermann K., Zipfel S., Enck P., Penders J. (2016). Weight gain in anorexia nervosa does not ameliorate the faecal microbiota, branched chain fatty acid profiles and gastrointestinal complaints. Sci. Rep..

[281] Yang J., Keshavarzian A., Rose D.J. (2013). Impact of dietary fiber fermentation from cereal grains on metabolite production by the fecal microbiota from normal weight and obese individuals. J. Med. Food.

[282] Baxter N.T., Schmidt A.W., Venkataraman A., Kim K.S., Waldron C., Schmidt T.M. (2019). Dynamics of Human Gut Microbiota and Short-Chain Fatty Acids in Response to Dietary Interventions with Three Fermentable Fibers. mBio.

[283] Rutting S., Xenaki D., Lau E., Horvat J., Wood L.G., Hansbro P.M., Oliver B.G. (2018). Dietary omega-6, but not omega-3, polyunsaturated or saturated fatty acids increase inflammation in primary lung mesenchymal cells. Am. J. Physiol. Lung Cell. Mol. Physiol..

[284] McKeever T.M., Lewis S.A., Cassano P.A., Ocké M., Burney P., Britton J., Smit H.A. (2008). The relation between dietary intake of individual fatty acids, FEV_1_ and respiratory disease in Dutch adults. Thorax.

[285] Atlantis E., Cochrane B. (2016). The association of dietary intake and supplementation of specific polyunsaturated fatty acids with inflammation and functional capacity in chronic obstructive pulmonary disease: A systematic review. Int. J. Evid. Based Healthc..

[286] Fulton A.S., Hill A.M., Williams M.T., Howe P.R., Coates A.M. (2015). Paucity of evidence for a relationship between long-chain omega-3 fatty acid intake and chronic obstructive pulmonary disease: A systematic review. Nutr. Rev..

[287] Ekroos K., Lavrynenko O., Titz B., Pater C., Hoeng J., Ivanov N.V. (2020). Lipid-based biomarkers for CVD, COPD, and aging–A translational perspective. Prog. Lipid Res..

[288] Liu D., Meister M., Zhang S., Vong C.-I., Wang S., Fang R., Li L., Wang P.G., Massion P., Ji X. (2020). Identification of lipid biomarker from serum in patients with chronic obstructive pulmonary disease. Respir. Res..

